# Genome-wide epigenetic modifications in sports horses during training as an adaptation phenomenon

**DOI:** 10.1038/s41598-023-46043-w

**Published:** 2023-11-01

**Authors:** Katia Cappelli, Samanta Mecocci, Andrea Porceddu, Emidio Albertini, Andrea Giontella, Arianna Miglio, Maurizio Silvestrelli, Andrea Verini Supplizi, Gianpiero Marconi, Stefano Capomaccio

**Affiliations:** 1 Department of Veterinary Medicine, University of Perugia, 06123 Perugia, Italy; 2 Sports Horse Research Center (CRCS), University of Perugia, 06123 Perugia, Italy; 3 Department of Agraria, University of Sassari, 06123 Sassari, Italy; 4 Department of Agricultural, Food and Environmental Sciences, University of Perugia, 06121 Perugia, Italy

**Keywords:** Biochemistry, DNA, Genetics, Agricultural genetics, Epigenetics, Epigenomics, Functional genomics, Gene expression, Gene regulation, Genome, Sequencing, Molecular biology, Epigenetics, Transcription

## Abstract

With his bicentennial breeding history based on athletic performance, the Thoroughbred horse can be considered the equine sport breed. Although genomic and transcriptomic tools and knowledge are at the state of the art in equine species, the epigenome and its modifications in response to environmental stimuli, such as training, are less studied. One of the major epigenetic modifications is cytosine methylation at 5′ of DNA molecules. This crucial biochemical modification directly mediates biological processes and, to some extent, determines the organisms' phenotypic plasticity. Exercise indeed affects the epigenomic state, both in humans and in horses. In this study, we highlight, with a genome-wide analysis of methylation, how the adaptation to training in the Thoroughbred can modify the methylation pattern throughout the genome. Twenty untrained horses, kept under the same environmental conditions and sprint training regimen, were recruited, collecting peripheral blood at the start of the training and after 30 and 90 days. Extracted leukocyte DNA was analyzed with the methylation content sensitive enzyme ddRAD (MCSeEd) technique for the first time applied to animal cells. Approximately one thousand differently methylated genomic regions (DMRs) and nearby genes were called, revealing that methylation changes can be found in a large part of the genome and, therefore, referable to the physiological adaptation to training. Functional analysis via GO enrichment was also performed. We observed significant differences in methylation patterns throughout the training stages: we hypothesize that the methylation profile of some genes can be affected early by training, while others require a more persistent stimulus.

## Introduction

The horse is an extraordinary athlete, shaped by evolution and human selection, with a marked aptitude for speed and endurance. Being prey, the horse needed to run at remarkable speed for considerable distances to escape predators. The horse's anatomy is, therefore, adapted to speed and stamina. Man has exacerbated these innate athletic skills through the selection that introduced dramatic phenotypic differences to Thoroughbred or Arab breeds^1,2^. For example, the race-oriented selection had a strong footprint on Thoroughbred, enhancing high-performance characteristics^3^ such as Type IIA muscle fiber percentage up to 80–90% and boosting mitochondrial density with the intense activity of oxidative enzymes^4,5^.

Genetics is just one piece of the puzzle, especially in sports: the full potential is reached only after proper athletic preparation.

The Thoroughbred is an early, fast-growing breed, and training usually begins at 15 months of age. One tangible aim in race-oriented training is to enhance the horse's ability to reach the highest speed before reaching VO_2_ max. In untrained Thoroughbreds, this limit is approximately 50 km/h, but it can be pushed up to 60 km/h after proper and intense training^6^. Training must be carefully planned since, while VO_2_ max and aerobic capacity should be achieved early in training sessions, skeletal muscle improvement can only be observed after 4–6 months. This physiological gap often causes long bone and soft tissue problems, especially in young subjects^7^.

The study of the modifications induced by the environment (i.e., by training) challenges the scientific community to consider another determination layer of complex traits: epigenetic changes. Only a few studies have been done in sport horse exercise epigenetics. Still, there is no doubt that, due to its influence on gene transcription, DNA methylation will necessarily modify an individual's response to an exercise stimulus^8,9^.

Epigenetic changes due to exercise can be transient, for example, after a single session of high-intensity aerobic exercise or stable^10^. Skeletal muscle cells can retain DNA methylations caused by stress (inflammatory state) during early childhood and adulthood. Moreover, studies assume that such modifications can be vertically inherited^11^. In addition, skeletal muscles can modify their epigenetic fingerprint even with short-term environmental stimuli, allowing future adaptations to the same stimuli; this can be referred to as muscle epi-memory^12,13^. Indeed, it has shown that 60 min of high-intensity training (75% VO_2_ max/maximum aerobic capacity) leads to induced acetylation of H3 histone in human skeletal muscle^12^; in addition, satellite cells can proliferate, self-renewing, and potentially transmitting epigenetic changes to the daughters^11^.

Methylation profiles change in response to physical activity in a dose-dependent, gene-specific, and tissue-specific manner. In skeletal muscle, long-term exercise (6 weeks of resistance training) leads to an increase in the number of differentially methylated regions^14^; at the same time, there is a decrease in methylation levels of metabolic genes^15^. It is also known that, after an initial resistance training program and subsequent detraining, a second period of resistance training is characterized by a higher frequency of de-methylated sites compared to the first workout, suggesting the potential for an "epigenetic memory" of hypertrophy in skeletal muscle^12,16^.

A few studies concerning the impact of exercise on DNA methylation have also been carried out in the horse, particularly in the Thoroughbred^8^, which show how this epigenetic modification has great relevance during exercise adaptation in terms of de-methylation and methylation of different genes. Increased transcription of key regulatory, metabolic, and myogenic genes appears to be an *early response* to exercise in humans and horses, important for mediating subsequent adaptations in skeletal muscle^8,17^.

In the innovative field of epigenetics, different methods are available to determine the methylation status of DNA samples. Methylation is considered a relatively stable marker, easily available, and with non-invasive sampling^18^.

Several techniques have been developed with different strategies, each with advantages and limitations; all technologies available today still have major limitations, such as sensitivity, specificity, accuracy, and data interpretation^19,20^.

Recently, methylation content sensitive enzyme double-digest restriction-site-associated DNA (ddRAD) technique (MCSeEd) has been developed^21^, a reduced-representation, reference-free, cost-effective approach for characterizing whole genome methylation patterns across different methylation contexts.

Therefore, the study aims to deepen the knowledge of the epigenetic response to exercise in Thoroughbred horses, monitoring their first three-month training period immediately after taming. The MCSeEd (Methylation-context-sensitive-enzyme-ddRAD) method, which is an evolution of the ddRADseq (double-digest-restriction-site-associated-sequencing) method^22^, was applied to compare white blood cell DNA methylation profiles of two-year-old horses at different stages of training (T0 = start of intense training; T30 = after one month of training; T90 = after three months of training). This allowed us to obtain, for the first time, indications of the epigenetic modifications occurring in the Thoroughbred under training.

## Results

The genomic DNA from the buffy coat of twenty (20) Thoroughbreds (gathered in bulks of 4) was used to assess the DNA methylation changes during the first training season, monitoring the horses for three months after a month of acclimatization. To this purpose, the MCSeEd technique was applied, sequencing the fragments generated by a coupled enzymatic digestion using methylation-sensitive and non-sensitive enzymes.

### Sequencing, mapping, and DMPs identification

Single-end reads were produced from the fifteen (15) libraries, and after demultiplexing and cleaning procedures as previously described, a total of 131,296,618 sequences were used for the MCSeEd bioinformatic pipeline. On average, 8.7 million reads per sample were produced (details in Table 1).

**Table 1:** Sequencing and mapping statistics per sample.

Sample	Input RAW	Uniquely mapped	% Uniquely mapped	Counted in annotation	% counted in annotation
T0_1	4,804,767	3,975,751	82.75	3,975,444	99.99
T0_2	7,739,825	6,474,930	83.66	6,474,330	99.99
T0_3	7,178,851	5,934,569	82.67	5,934,111	99.99
T0_4	9,500,000	7,948,323	83.67	7,947,652	99.99
T0_5	8,851,915	7,356,630	83.11	7,355,924	99.99
T30_1	10,861,335	8,974,483	82.63	8,973,573	99.99
T30_2	9,364,504	7,849,062	83.82	7,848,317	99.99
T30_3	6,016,412	4,970,863	82.62	4,970,318	99.99
T30_4	9,357,060	7,839,397	83.78	7,838,874	99.99
T30_5	7,352,074	6,116,479	83.19	6,115,909	99.99
T90_1	12,164,051	10,077,752	82.85	10,076,735	99.99
T90_2	12,470,936	10,477,653	84.02	10,476,511	99.99
T90_3	7,928,217	6,572,276	82.90	6,571,669	99.99
T90_4	9,993,804	8,357,337	83.63	8,356,319	99.99
T90_5	7,712,867	6,410,731	83.12	6,409,842	99.99

Uniquely mapped reads on EquCab3 were in the proportion—on average between samples—of 83.2% of the input sequences. Virtually all of them were then reassigned to the experiment-wise annotation. After robust filtering and normalization, 94,957 loci were input to methylKit for downstream analysis. This number was further reduced considering only those covered by at least ten reads and statistically different throughout the time points (DMPs, differently methylated positions) with a false discovery rate (FDR) < 0.05, resulting in 30,773 loci used for DMRs (differently methylated regions) discovery.

**Figure 1:**
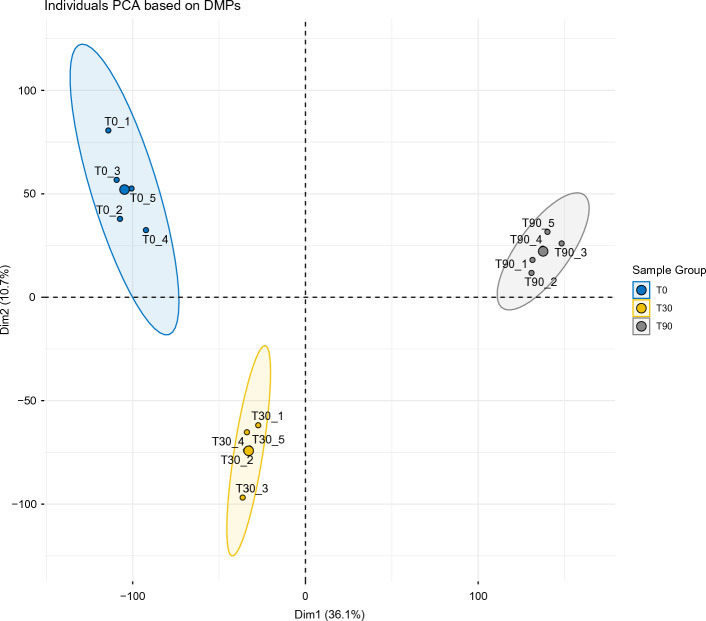
Plot of components 1 and 2 identified by the PCA analysis. Clear clustering of samples in homogeneous groups for time points is evident. Sample groups are indicated in the legend. Correct library separation can also be appraised in the dendrogram shown in Supplementary Fig. S1.

To verify filtering and experimental design soundness, a principal component analysis (PCA) was carried out (Fig. 1), obtaining a separate clustering of the bulks belonging to the same time (T0, T30, and T90) with the first two dimensions absorbing more than 40% of the total variance. The correct separation of libraries can be appraised also in the dendrogram shown in Supplementary Fig. S1.

### DMR analyses

The DMRs were identified starting from the DMPs of pairwise comparisons between the three-time points through the iterative procedure previously described. Results are reported in Supplementary Tables S1, S2, and S3.

From the DMP clustering, the optimal width of the window in terms of bp used for DMR detection was 700 bp, 1100 bp, and 1300 bp, containing 126, 624, and 353 DMRs for T30-T0, T90-T0, and T90-T30 comparisons, respectively (Table 2).

**Table 2:** Number of DMPs and DMRs in the three comparisons with relative modulation.

Comparison	DMPs	Number of de-methylated DMPs	Number of methylated DMPs	DMRs	Number of de-methylated DMRs	Number of methylated DMRs
T30 vs. T0	256	46	210	126	23	103
T90 vs. T0	1275	444	831	624	218	406
T90 vs. T30	718	356	362	353	174	179
Total	2249	846	1403	1103	415	688

The genomic coordinates of the DMRs were intersected with the Ensembl annotation, obtaining a total of 733 genes involved. Table 3 gives each comparison's number of genes and the methylation state. In Supplementary Table S4, this information is available for single genes comprising the DMR site within the gene (gene body or regulatory region).

**Table 3:** Number of differentially methylated genes among the three comparisons, with relative quantities of de-methylated and methylated genes.

Comparison	Number of genes	Number of de-methylated	Number of methylated
All	733	275	458
T30 vs. T0	86	10	76
T90 vs. T0	424	138	286
T90 vs. T30	223	127	96
*Early response*	70	6	64
*Mid response*	207	116	91

Using all comparisons, we tried to isolate a group of genes, hereafter named “*early response*”, that could be influenced by training already at T30 (early stages of training); “*mid response*”, conversely, are those genes that are not affected at T30, but in the following stages. A Venn diagram clarifying the rationale behind this diversification is reported in Supplementary Figure S2. The genes present in the T90–T0 comparison, which are neither found in *mid response* nor *early response*, can be considered the ones whose modification in methylation is present continuously throughout the whole period, increasing or decreasing progressively.

### Functional analyses and gene ontology

To highlight biological processes, molecular functions, and cellular compartments mainly involved in the response to exercise and training during the different stages, a Gene Ontology (GO) enrichment analysis was carried out. The complete results of the functional analysis for differentially methylated genes from all the comparisons (T30 vs. T0, T90 vs. T0, T90 vs. T30, *early* and *mid* *response*) are reported in Supplementary Table S5. Table 4 lists the first fifteen most significant (lower corrected *p-*value) terms. While the most represented terms for *early response* modulated genes were related to protein regulation and cell signaling, *mid response* genes were mainly involved in cell communication, synaptic transmission, and regulation of transmembrane transporter activity. The genes that were differentially methylated in the whole training time frame showed enrichment in categories related to blood circulation, cardiac functions, and immune regulation.

**Table 4:** The first fifteen enriched GO terms (lowest adjusted p-value by Bonferroni correction) resulted from the ClueGO algorithm for differentially methylated genes during *early response*, *mid* *response*, and whole period.

Time frame	ClueGO enriched term	Corrected p-value	Nr. genes
*Early response*	Protein maturation	0.00047	7
	Positive regulation of cysteine-type endopeptidase activity involved in apoptotic process	0.00828	3
	Neutral lipid metabolic process	0.00828	3
	Intrinsic apoptotic signaling pathway in response to endoplasmic reticulum stress	0.01183	3
	Acylglycerol metabolic process	0.01626	3
	Negative regulation of binding	0.01718	4
	Negative regulation of binding	0.01718	4
	Specification of symmetry	0.02349	3
	Negative regulation of transmembrane receptor protein serine/threonine kinase signaling pathway	0.02349	3
	Cargo receptor activity	0.02544	3
	Amyloid fibril formation	0.02884	3
	Camera-type eye morphogenesis	0.02906	3
	Regulation of BMP signaling pathway	0.02971	3
	Activation of cysteine-type endopeptidase activity involved in apoptotic process	0.02999	3
	Maintenance of protein location	0.02999	3
*Mid response*	Regulation of cation transmembrane transport	0.00394	13
	Lytic vacuole membrane	0.00519	13
	Lysosomal membrane	0.00519	13
	Regulation of ion transmembrane transport	0.01568	14
	Vacuolar membrane	0.01767	13
	Regulation of ion transmembrane transporter activity	0.02176	10
	Regulation of transmembrane transporter activity	0.03096	10
	Anterograde trans-synaptic signaling	0.03113	17
	Chemical synaptic transmission	0.03113	17
	Trans-synaptic signaling	0.03600	17
	Extrinsic component of cytoplasmic side of plasma membrane	0.04077	6
	Dendritic spine	0.04541	8
	Synaptic signaling	0.04750	17
	Regulation of transporter activity	0.04772	10
	Regulation of ion transport	0.04875	16
Whole period	Regulation of blood circulation	0.00004	22
	Regulation of heart contraction	0.00006	19
	Cardiac conduction	0.00008	14
	Neuron projection guidance	0.00009	21
	Wnt signaling pathway	0.00035	28
	Lysosomal membrane	0.00042	23
	Modulation of chemical synaptic transmission	0.00054	24
	Vacuolar membrane	0.00058	24
	Epithelial cell migration	0.00058	19
	Regulation of epithelial cell migration	0.00062	16
	Transmembrane transporter complex	0.00086	19
	Regulation of cation transmembrane transport	0.00101	21
	Synapse organization	0.00102	23
	Dendritic spine	0.00103	14
	Calcium channel complex	0.00115	8

### Gene expression

**Figure 2:**
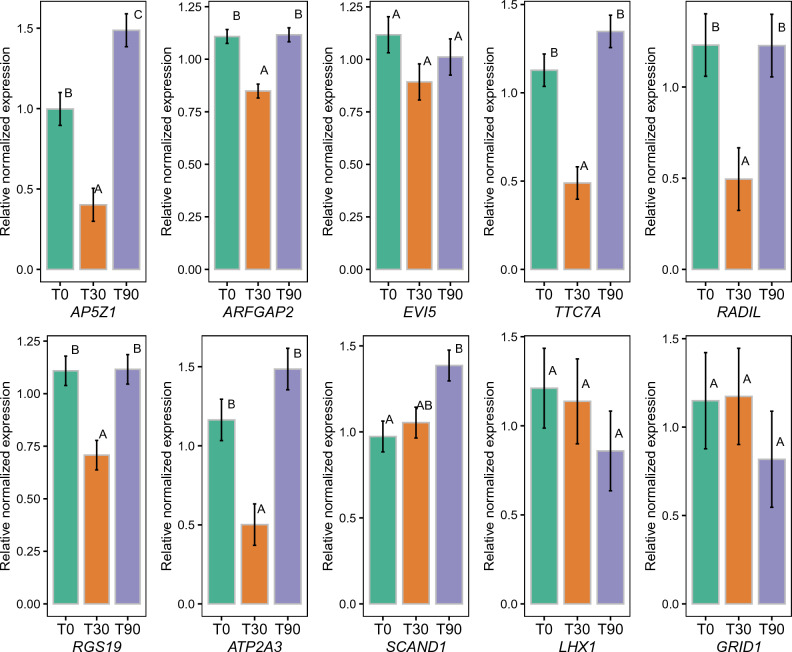
Bar plot with relative normalized gene expression of tested genes among the three-time points. Different letters within each box indicate statistical significance (p < 0.05).

To validate the MCSeEd results, 10% of the highest differentially methylated genes among time points were checked for expression in silico in peripheral blood mononuclear cells (PBMC) racehorse transcriptomes and in a human PBMC atlas. The results obtained are reported in Supplementary Table S6. Moreover, some of these genes, which were particularly interesting from the functional analysis, were tested for their gene expression through RT-qPCR (Fig. 2). All the tested genes were expressed in PBMCs. Low expression levels were found for *GRID1* and *LHX1*, which, indeed, were not modulated during training.

Moreover, the agreement between the methylation results and gene expression analysis was checked (Table 5), and results were completely in line when both tests were statistically significant. Indeed, the lack of agreement exclusively occurred when one of the two tests did not reach statistical significance.

**Table 5:** Agreement between MCSeEd and qRT-PCR results.

Gene	Comparison	MCSeEd outcome	RT-qPCR outcome	Agreement
*AP5Z1*	T30 vs. T0	N.S.	Down-regulation	
	T90 vs. T0	De-methylation	Up-regulation	Yes
	T90 vs. T30	De-methylation	Up-regulation	Yes
*ARFGAP2*	T30 vs. T0	N.S.	Down-regulation	
	T90 vs. T0	De-methylation	N.S.	
	T90 vs. T30	De-methylation	Up-regulation	Yes
*EVI5*	T30 vs. T0	N.S.	N.S.	
	T90 vs. T0	De-methylation	N.S.	
	T90 vs. T30	De-methylation	N.S.	
*GRID1*	T30 vs. T0	Methylation	N.S.	
	T90 vs. T0	De-methylation	N.S.	
	T90 vs. T30	N.S.	N.S.	
*LHX1*	T30 vs. T0	Methylation	N.S.	
	T90 vs. T0	N.S.	N.S.	
	T90 vs. T30	N.S.	N.S.	
*RADIL*	T30 vs. T0	N.S.	Down-regulation	
	T90 vs. T0	De-methylation	N.S.	
	T90 vs. T30	De-methylation	Up-regulation	Yes
*ATP2A3*	T30 vs. T0	Methylation	Down-regulation	Yes
	T90 vs. T0	Methylation	N.S.	
	T90 vs. T30	N.S.	Up-regulation	
*RGS19*	T30 vs. T0	Methylation	Down-regulation	Yes
	T90 vs. T0	N.S.	N.S.	
	T90 vs. T30	N.S.	Up-regulation	
*SCAND1*	T30 vs. T0	N.S.	N.S.	
	T90 vs. T0	De-methylation	Up-regulation	Yes
	T90 vs. T30	De-methylation	N.S.	
*TTC7A*	T30 vs. T0	N.S.	Down-regulation	
	T90 vs. T0	N.S.	N.S.	
	T90 vs. T30	De-methylation	Up-regulation	Yes

Statistical significance of modulated methylation (de-methylated–methylated) or gene expression (up-regulated–down-regulated) is reported among the comparisons (T30 vs. T0, T90 vs. T0 and T90 vs. T30). N.S.: not statistically supported. The “Agreement” column was compiled only when both analysis outcomes were statistically supported.

*AP5Z1* was de-methylated in T90 compared to T0 and T30, and its expression increased in the same comparisons. This gene was also down-regulated in T30 with respect to T0, although no differences in methylation levels were found, indicating a possible post-transcriptional regulation mechanism. *ARFGAP2* was de-methylated in T90 compared to T30 and T0, but the reduction in gene expression was significant only in T90 vs. T30. Also, in this case, a downregulation in gene expression was detected in T30 compared to T0, while no modulation in methylation was statistically significant. For *EVI5*, the de-methylation in T90 compared to T0 and T30 did not result in significant gene expression modifications, nor did the methylation in T90 vs. T0 for *RADIL*. Moreover, the expression of *RADIL* was lower in T30 compared to T0, although without differences in methylation, while the de-methylation in T90 vs. T30 matched with the gene expression increase. The *RGS19* and *ATP2A3* methylation in T30 compared to T0 generated a decrease in gene expression, as expected, while the up-regulation of the same genes in T90 vs. T30 was probably not induced by a de-methylation which was not statistically supported, as well as for *TTC7A* down-regulation in T30 vs. T0. Moreover, *TTC7A* was up-regulated in T90 compared to T30 and de-methylated in the same comparison, concordantly. As expected, the de-methylation of *SCAND1* in T90 (either vs. T0 or T30) resulted in an increased expression in T90 (compared to T0). For the low-expressed genes *LHX1* and *GRID1*, the modulations in methylation observed were not followed by differences in gene expression, probably for the low basal expression due to other regulatory mechanisms.

## Discussion

Adaptation to exercise and training triggers various responses mediated by several molecular signaling events with complex spatial and temporal interactions involving the genome at different levels based on the stimulus's intensity, duration, and frequency. One aspect of this fine machinery is the epigenetic regulation, known to modulate gene expression with no sequence variation and to some extent heritable^23^.

Emerging evidence shows that physical activity influences DNA methylation in humans^24^ and that trainability depends not only on the genetic code but also on epigenetic signals such as DNA methylation and histone modifications. The most accessible tissue where epigenetic modifications (especially global methylation profile) can be investigated is the blood. However, many challenges must be faced because patterns change rapidly depending on the intensity and duration of the exercise^25^. Investigating methylation changes during a long training period, especially in never-trained athletes, could give more consistent results than comparing subjects of different ages and training.

Considering these assumptions, our study evaluated methylation changes in the white blood cell DNA of Thoroughbred horses at their first 90 days of training, identifying differentially methylated regions (DMRs) from differentially methylated positions (DMPs) through the iterative procedure described (Table 2). A list of Ensembl-annotated genes that matched at least one DMR was produced (Supplementary Table S4). Then, the 10% of top methylated and de-methylated genes in each comparison were identified and used for Gene Ontology (GO) categories enrichment analysis. For these genes, the expression in the same cells in which methylation was assessed was investigated through RT-qPCR. Results showed that all chosen genes were expressed in blood, and their modulation mostly agrees with methylation status, confirming that MCSeEd is a valid approach to exploring genome-wide epigenomic changes (Table 5).

### Differentially methylated genes

Studies in humans and animal species (rat and horse)^8,26^ report that most genes involved in this regulation are mainly ascribed to muscle growth and differentiation, innervation, and synaptic-related functions. In contrast, metabolic regulation is engaged to a lesser extent.

In our system, the GO enrichment analysis (Table 4, Supplementary Table S5) of the differently methylated genes showed cell signaling proteins, ion channels, innervation and synaptic transmission, heart conduction, and contraction as the most represented terms.

It is worth noting that muscle growth does not depend only on the development and differentiation of muscle cells, which is necessary for hypertrophic and/or reparative processes induced by exercise, but also requires the innervation of motor neurons and the proliferation of blood vessels^8^.

### “Early response” to exercise

The first training comparison (T30 vs. T0) (Table 2) revealed a prevalence of methylated regions (103 DMRs methylated vs. 23 de-methylated) that, once crossed with the Ensembl annotation pinpointed, respectively, 76 and 10 genes (Supplementary Tables S4 and S6). Considering the *early response* gene subset, thus excluding those in common with T90–T0 comparison (Supplementary Fig. S2), the prevalence of methylation is even more evident (64 methylated vs. 6 de-methylated genes).

This is in agreement with what observed in humans: exercise and training initially lead to increased methylation of specific genes, especially those related to transcriptional activity, while if the training exceeds eight weeks, a general de-methylation is observed, especially for those genes related to metabolic and actin-cytoskeleton pathways^27^.

#### Functional analyses

Concerning the GO enrichment analyses of the *early response* genes, GO terms related to protein regulation and cell signaling were found (e.g., “protein maturation”, “maintenance of protein location growth and development and cell signaling”, “negative regulation of binding”, “negative regulation of transmembrane receptor protein serine/threonine kinase signaling pathway”; Table 4, Supplementary Table S5).

If we take into account the whole gene set in T30–T0 comparison, additional GO terms, such as “regulation of blood circulation”, “cardiac conduction”, “regulation of heart contraction”, and “immunological synapse”, were statistically significant. Genes described by these categories are typically ascribed to other organs, but they can be expressed in blood cells and possibly with specific functions related to exercise.

### Gene-expression analyses

Most *early response* differentially methylated genes were also differentially expressed in RT-qPCR experiments and related to exercise-activated pathways.

In detail, for example, a gene also known to be methylated as a function of insulin and glucose levels in human athlete^28^, calcium pump *ATP2A3,* was found in several enriched categories and among methylated genes (Supplementary Tables S4 and S6). Moreover, as seen in RT-qPCR, the *ATP2A3* methylation in T30-T0 generated a gene expression reduction (Fig. 2, Table 5).

Another gene enriched in several GO categories and methylated in the T30-T0 comparison is the regulator of G protein signaling (*RGS*). The members of the RGS family are molecules acting on the G protein-mediated signal as negative regulators. This gene is also expressed in blood cells and regulated by hormones, cytokines, and Ca^2+^ oscillations^29^. This G protein is crucial for bone cell growth and differentiation, and this gene is frequently expressed in cardiac hypertrophy and heart attack^30^. This could mean that the stimulation of the musculoskeletal system and immune cells given by training could be driven by epigenetic mechanisms such as the methylation status modulation of key pathway genes for different target tissues.

Human data reveal no expression for the *RGS* family in PBMCs (Supplementary Table S6); in horses, it is expressed both at rest and immediately after the race. Moreover, in this experiment, the *RGS19* gene, identified among the most methylated ones, is expressed in leukocytes and is down-regulated in T30 compared to T0, in agreement with its epigenetic regulation (Supplementary Table S4, Fig. 2).

Again, *GRID1 is* an interesting gene among the most methylated in *early response*, encoding a subunit of glutamate receptor channels. These channels mediate most of the fast excitatory synaptic transmission in the central nervous system and play key roles in the synaptic plasticity^31^. Modulation of glutaminergic synapses at the level of postsynaptic density by voluntary exercise was also highlighted in mice. Therefore the glutamatergic system could be a target of modulation through regular physical activity^32^. This finding is unexpected as no expression was found in the human or horse atlas; however, it is always expressed in our system but not modulated.

Since its function is strongly related to exercise, we can speculate that its methylation change may have a biological meaning (Supplementary Tables S4 and S6).

The LIM homeobox 1 gene (*LHX1*), an *early response* one, is involved in the locomotor apparatus regulating the differentiation of Purkinje cells which are responsible for fine control of movements^33^; it is required for temperature resistance of the nervous system clockworks^34^, and it was recently found as a novel pancreatic islet regulator contributing to normal glucose homeostasis and glucagon-like peptide 1(Glp1)^35^. Intriguingly, in a study by Pereira et al.^36^, this gene was associated with race performance in racing Quarter Horse. Also, in this case, *LHX1* does not appear to be expressed in humans or horse PBMCs, while from RT-qPCR experiments, it is expressed but not modulated across sampling time points.

### “Mid response” to exercise

Focusing on the late phase after 90 days of training is also interesting. Indeed, in human athletes studies, it is shown that DNA methylation changes in response to exercise are a dynamic process activated in the early phase of gene expression; residual DNA methylation changes are retained after the training stimulus is gone, indicating that these modifications are accumulated over multiple exercise sessions^37^.

In our study, the comparison T90–T0 pinpointed 424 genes from 624 DMRs (Table 2) with 138 de-methylated genes vs. 286 methylated, resulting in an increase of the de-methylated genes with respect to the first part of training. In this comparison, all the modulated genes are considered from the beginning to the end of the training.

Of these, 205 (91 are methylated and 114 de-methylated) were classified as *mid response* genes (Supplementary Fig. S2). There seems to be a preference for de-methylation as training progresses, as this subset includes 91 out of 138 de-methylated genes (66%) and only 114 out of 286 methylated genes (40%). This is coherent with human athlete study findings, where a higher number of de-methylated sites was associated with hypertrophy in the muscle following a repeated stimulus of 7 weeks^13^. This indicates that changes in DNA methylation could be related to exercise training and that, as the time of training increases and optimal athletic condition approaches, de-methylation of some specific genes also increases.

#### Functional analyses

From the enrichment analysis on the *mid response* gene set, the most represented terms were “cell communication”, “signal transmission”, “synaptic transmission” (BinGo analyses) and “synaptic signaling”, “regulation of transmembrane transporter activity” (ClueGo analyses) (Supplementary Table S5).

#### Gene-expression analyses

Also, among the *mid response* genes, some of the most de-methylated genes were RT-qPCR tested (*SCAND1*, *RADIL*, *EVI5*, *AP5Z*, *TTC7A*, *ARFGAP2*) (Supplementary Table S4). These genes encode for transcription factors, small GTPases-related genes for membrane transport processes, or other signal mediators, which fall into the GO-enriched categories for this comparison.

The* SCAND1 *gene, encoding for a transcription factor with DNA-binding activity, is one of the most de-methylated in mid response. It has been detected as a co-expressed gene in a network analysis from muscle and neutrophil tissues: this suggests the preservation of robust gene expression profiles among a blood leukocyte subpopulation and the skeletal muscle in response to physiological stresses^38^.

This supports the idea that neutrophil gene networks may help track physiological and pathological changes in the muscle tissue, especially for genes that are functionally related to post-translational signaling mechanisms such as acetylation^38^. *SCAND1* is expressed in human and horse PBMCs and our system with concordance between the methylation change and the relative gene expression.

*RADIL* gene that encodes for Ras-associated and dilutes domain-containing protein with a GTPase binding activity and is involved in the regulation of cell–cell adhesion in endothelial cells^39^, also matched its de-methylation in T90–T30 with the gene expression increase in RT-qPCR.

Also, EVI5, a product of the *EVI5* gene*,* acts as a GTPase Activating Protein (GAP) for the Rab family GTPases that regulate membrane traffic. Moreover, the *ARFGAP2* gene encoded for Arf proteins, small GTPases that are key elements of downstream signaling pathways regulating multiple effector proteins and functional responses of cells. Arf protein regulates polymorphonuclear neutrophil functions such as superoxide production, degranulation, and chemotaxis^40^ in blood. Both genes are expressed in human and horse PBMCs, and in the horse buffy coat analyzed in our study. T90–T30 de-methylation of *ARFGAP2* corresponded to an increased expression in RT-qPCR, while T90–T30 de-methylation of *EVI5* did not correspond to a significant gene expression modulation; however, the regulation of transcription does not depend only on the state of DNA methylation.

The expression of the *AP5Z1* gene, encoding a crucial protein for intracellular cell trafficking by controlling features such as cell development, signal transduction, apoptosis, and proliferation pathways^41^, is instead modulated accordingly with its methylation state in different libraries. Like the *TTC7A* gene (up-regulated in leukocytes of the T90 library compared to T30 and de-methylated in the same comparison) that encodes a scaffolding protein, facilitating the synthesis of PI4-phosphate (PI4P) essential for promoting transport of secretory proteins^42^.

In conclusion, in this study, we applied, for the first time, the methylation content sensitive enzyme ddRAD (MCSeEd) technique to animal blood cells and observed substantial and significant differences in methylation patterns during early training stages with insights also in mid and late training stages where more persistent stimuli are required.

The methylation changes observed are dramatically high and largely a consequence of the incrementing training, although we cannot completely rule out other environmental stressors, such as weather conditions. Some authors report, although with not so compelling results, that seasonality and mean temperature during short-, medium-, and long-term exposures can mildly modify the methylation status of a genome^43–45^. In our case, the THI (temperature humidity index) was monitored to evaluate its changes throughout the time span of the experiment^46^. The minimum THI for all the days was lower than 70, which is the critically recognized threshold in farm animals to pinpoint heat stress^47^. To limit the possible changes induced by high environmental temperature, both blood sampling and training were carried out early in the morning (6:30 AM), a time which is not associated with acute heat stress (48.1 < THI < 64, Table 2 in^46^); in the hottest hours, animals were kept under indoor-temperature-controlled stables. The methylation changes associated with temperature previously observed in literature were on a different scale compared to those we found, both in terms of methylation levels and the number of implicated genes that were different and also on other functional categories; only 4 out of 446 (0.9%) genes here differentially methylated have already been associated with temperature^44,45^. Moreover, even though a change does exist, spring and summer—our monitored time frame—appear to exert similar outcomes^43^.

All considered, the environmental footprint on methylation due to temperature and humidity is very likely masked by the more impactful effect induced by training.

Our results indicate that the genome-wide approach is mandatory to fully understand and characterize a complex phenomenon, such as the response to training.

We have confirmed that genes belonging to GO categories, typically linked to other organs, can be expressed in blood cells with specific functions also related to exercise.

Indeed, we have highlighted that genes—and therefore relative functions and GO categories—are far from fully characterized, especially those expressed in blood tissue that mimics the systemic response.

The results of this research could be prodromal to the characterization of the epigenetic response induced by exercise in the horse athlete, to enhance training schedules, improve performances, and better respect animal health and welfare.

## Methods

### Animals enrolled

For this study, 20 Thoroughbreds (8 males and 12 females) with a mean age of two years (Supplementary Table S7), clinically healthy, and never trained for flat racing were recruited. For sampling, an aliquot from the blood sampled for routine controls to assess the health of the animals during the training season, allowed by the Italian Horse Racing Board and performed by the authorized veterinary practitioner, was taken^48^. All animals were enrolled in the study after the owners' and trainers' written informed consent in compliance with the Italian Regulation D.L. 116/1992. Anyway, the animal care procedures were compliant with the European recommendations (Directive 2010/63/EU) for the protection of animals used for scientific purposes, and the study is reported in accordance with the ARRIVE guidelines.

All the horses were managed in the same stable with individual housing with natural temperature and photoperiod. The training program is summarized in Table 6 and was performed for each horse from Monday to Saturday. The two months before the training start (T0) were used to acclimate the animals.

**Table 6:** Standard daily training program completed by each horse involved in the study.

Acclimation	T -30	T0	T30	T60	T90
15’ Walk 10’ Trot Rest 10’ Trot Walk	15’ Walk 10’ Trot 3’ Canter	15’ Walk 10’ Trot 6’ Canter Tuesday: 1’ Gallop	15’ Walk 10’ Trot 6’ Canter Tuesday: 2’ Gallop	15’ Walk 10’ Trot 6’ Canter Tuesday: 3’ Gallop	15’ Walk 10’ Trot 6’ Canter Tuesday: 4’ Gallop

At the end of the experimental period, all subjects participated in one or more competitions; none showed a poor performance syndrome during the study or competition period. Weather conditions (temperature and relative humidity) were monitored during the whole period of training, collecting data at a weather station 6.6 km away from the training center at noon and calculating the temperature humidity index (THI) as reported in our previous study^46^ where samples of the same cohort were used.

### Sampling

The sampling activity was carried out once a month, from March 2018 to July 2018, at 6:30 AM before training and feeding, while at T90, the collection was carried out before the race. The experimental period was divided into five times, thirty days apart (T-30, T0, T30, T60, and T90). March (T-30) was considered to be the month in which the animals started the light gallop; April (T0) was the first month of training with racing simulation (gallop). From April to July, the training was incremental (see details in Table 6). Blood samples were collected from the jugular vein in Vacutainer tubes (10 ml; Terumo Corporation, BD; Tokyo, Japan) with EDTA. Samples at times T0, T30, and T90 were used to evaluate DNA methylation changes.

### DNA purification, library construction, and sequencing

**Figure 3:**
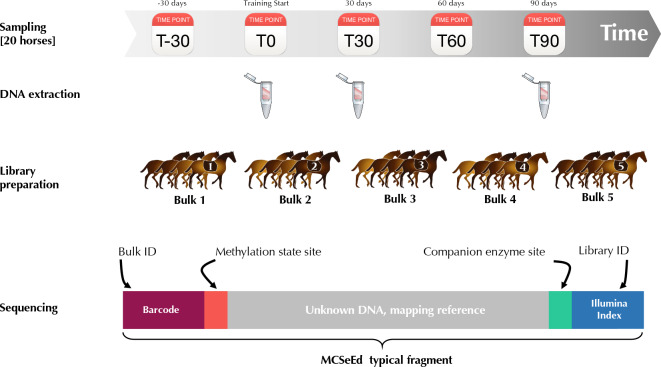
Scheme of the experimental design from sampling to obtain bulks and respective libraries, sequencing, and bioinformatics analysis.

The genomic DNA was extracted from the buffy coat with GenElute ™ Mammalian Genomic DNA Miniprep kit (Sigma-Aldrich, St. Louis, Missouri, USA) and subsequently quantified with a NanoDrop2000 spectrophotometer (Thermo Fisher Scientific, Waltham, MA, USA) and Qubit fluorometer (Thermo Fisher Scientific, Waltham, MA, USA). Successively samples were grouped into pools of 4 subjects at each time point of sampling, obtaining five different bulks (Fig. 3, Supplementary Table S8). We proceeded with bulks to mitigate individual genetic variation and reduce costs.

The library set-up protocol was performed according to Marconi et al.^21^ with some modifications, as described below. Before digestion, five methylation enzymes were evaluated for the best restriction profile for CG methylation context: *Aci*I, *Acl*I, *Age*I, *BsrF*I, and *BstB*I, with *Mse*I as a companion enzyme. To define the expected restriction pattern of an enzyme combination, the genome was scanned in silico*,* calculating the size distribution of the restriction fragments with the optimal length range (200–700 bp) effectively captured by MCSeEd genomic libraries. *Aci*I was chosen since it presented the highest number of suitable fragments (Supplementary Table S9).

For each library, 150 ng of DNA were double-digested with a combination of the two enzymes (5U of *Aci*I and 5 U of *Mse*I restriction enzymes, New England BioLabs, Ipswich, US), in the presence of 2 μM of unique barcoded adapter, 2 μM of unique common Y adapter, 1 U of T4 DNA ligase (Thermo Fisher), 0.2 mM ATP and 1 × RL buffer (5 × CutSmart Buffer, New England Biolabs, 25 mM DTT, Invitrogen) for a final volume of 50 μL.

The libraries were then pooled, as reported in the sequencing experimental design (Supplementary Table S8, purified using magnetic beads (Agencourt AMPure XP; Beckman Coulter, MA, USA), size selected by gel electrophoresis and purified using QIAquick Gel Extraction kits (Qiagen, Aarhus, DK) for fragments in the range of 200 bp to 700 bp. Size-selected libraries were quantified using a fluorometer (Qubit; Agilent Life Technologies, Santa Cruz. CA, USA), and a normalized DNA amount (15 ng) was amplified with a primer that introduced an Illumina Index (at the Y common adapter site) for demultiplexing. Following PCR with uniquely indexed primers, multiple samples were pooled. PCR enrichment was performed as previously described^21^. Amplified libraries were purified with magnetic beads (AMPure; Beckman Coulter, Brea, CA, USA) and then quantified (Qubit and Bioanalyzer 2100: Agilent Life Technologies). The grouped libraries were pooled in equimolar amounts, and the final library was Illumina-sequenced using 150-bp single-end chemistry.

### Bioinformatic analyses

Briefly, the MCSeEd pipeline follows this rationale. When a reference genome is available^49^, sequences are mapped on it, and alignment coordinates are saved in an “experiment-wise annotation” that allows creating a count matrix where rows are methylation-affected loci and columns are samples. The count file is then filtered and processed to ultimately obtain the differentially methylated positions (DMPs), according to the following steps: (i) standardization of libraries; (ii) filtering based on the coverage with at least ten reads per locus; (iii) calculation of the relative levels of methylation in each locus; (iv) parsing of datasets for methylKit R package usage^50^. In-depth details on the technical procedure are available in Marconi et al.^21^.

DMPs were therefore identified as sites that showed significant differences in the methylation levels between the treatments, using logistic regression as implemented in methylKit. The DMPs were called following the methylKit manual best practices. These positions were used to calculate the window within is possible the identification of a *differentially methylated region* (DMR) where at least two DMPs concordant (de-methylated, methylated) and statistically significant (FDR < 0.05) rely on. To achieve optimal DMR identifications, we maximized the number of DMRs in a series of adjacent sliding windows with an iterative procedure considering different window lengths (100 bp to 2000 bp with 100 bp pace).

The bioinformatics pipeline is available and maintained at https://bitbucket.org/capemaster/mcseed/src/master/.

### Differentially methylated genes

To obtain a gene list with methylation level modulation during training, we produced a BED format file of our DMRs that served as input, together with the complete annotation of EquCab3, Ensembl 101, downloaded from Biomart (http://www.ensembl.org/biomart/), to the *bedtools* utility (*intersect* function). Both the gene body and regulatory regions, 2.5 kb upstream of the transcription start sites (TSSs) for strand + genes and 2.5 kb downstream of the transcription termination site (TTS) for strand—genes, were considered for the intersection.

### Enrichment analyses

The gene list from the previous step was the base information for an enrichment analysis using Gene Ontology (GO) categories via BinGO^51^ and ClueGO^52^, both implemented in the Cytoscape suite^53^. These two pieces of software use different algorithms to reveal the enrichment of the GO terms, and we decided to pursue a conservative approach with a “consensus analysis*”*; only GO terms belonging to the categories "biological processes", "molecular functions", and "cellular compartments" statistically significant (FDR < 0.05) in both analyses were considered and discussed.

### Gene expression assessment

To validate methylation status/effects, the most modulated genes found with the highest differences in methylation among time points were selected (10% most methylated and 10% most de-methylated within each comparison). The expression levels of these genes were checked in our RNA-seq data of peripheral blood mononuclear cells (PBMCs) of racehorses at rest and after gallop race^54^ and, to further consolidate our findings, in a human PBMC gene expression atlas (EMBL-EBI Expression Atlas).

The expression levels are reported as mean transcript per million (TPM) of the five horses (Bio-Project PRJNA605934, with name sequences and accession numbers from S10B-SAMN14082184 to S9G-SAMN14082193) and the four human PBMC cell lines (HPSI0813er-fpdj, HPSI0813er-fpdl, HPSI0813er-fpdm, and HPSI0813er-fpdr).

Moreover, the relative gene expression of some of these genes (see list in Supplementary Table S10) was assessed through RT-qPCR. Total RNA was extracted from the buffy coat of the 20 horses for T0, T30, and T90, using Invitrogen™ TRIzol™ Plus RNA Purification Kit (Thermo Fisher Scientific, Waltham, MA, USA), following the manufacturer’s instructions. RNA extraction was assessed using a NanoDrop2000 Spectrophotometer (Thermo Fisher Scientific, Waltham, MA, USA). As for methylation assessment, samples were pooled into five bulks, each containing the RNA of the same four horses mixed for DNA.

The same amount of RNA for each bulk (1 µg) was reverse-transcribed using the SuperScript® VILO IV TM Master Mix (Thermo Fisher Scientific, Waltham, MA, USA), following the manufacturer’s guidelines. The cDNA was diluted in nuclease-free water 1:10, and the amplification was performed on CFX96™ Real-Time System (Bio-Rad, Milan, Italy) following a protocol developed in previous studies^55^. The succinate dehydrogenase complex flavoprotein subunit A (*SDHA*) and hypoxanthine phosphoribosyltransferase 1 (*HPRT*) are optimal reference genes for blood cells in horses and were used to normalize target gene expression levels^56,57^. Target gene primers were designed through the Primer3 online platform (https://primer3.ut.ee, accessed on 30th October 2023) and placed in different exons or at exon-exon junctions to avoid biases due to genomic DNA amplification. Supplementary Table S10 reports primer pair sequences for tested genes: SCAN domain containing 1 (*SCAND1*), glutamate ionotropic receptor delta type subunit 1 (*GRID1*), LIM homeobox 1 (*LHX1*), regulator of G protein signaling 19 (*RGS19*), rap associating with DIL domain (*RADIL*), ecotropic viral integration site 5 (*EVI5*), adaptor related protein complex five subunit zeta 1 (*AP5Z1*), tetratricopeptide repeat domain 7A (*TTC7A*) and ADP ribosylation factor GTPase activating protein 2 (*ARFGAP2*). The relative normalized expression was calculated through the 2^−∆∆Ct^ method, and statistical significance for differential gene expression was assessed in the R environment fitting a linear model with the *nmle* (https://cran.r-project.org/web/packages/nlme/) function setting bulks as random effect^58^. After normal distribution checking by the Shapiro–Wilk test, one-way ANOVA was applied, and the “emmeans” package (https://CRAN.R-project.org/package=emmeans) was used as post-hoc for pairwise comparisons between the three-time points, using compact letter displays (CLD) to highlight significance (*p* < 0.05).

### Supplementary Information

Supplementary Information.

## Data Availability

The data related to this article can be obtained in the Supplementary materials; contact the corresponding author dr. Samanta Mecocci (e-mail: samanta.mecocci@unipg.it) for other requirements.
