# Supplementary material for: Genome-wide epigenetic modifications in sports horses during training as an adaptation phenomenon
Source: Sci Rep. 2023 Nov 1;13:18786. doi: 10.1038/s41598-023-46043-w (PMC10620398; doi:10.1038/s41598-023-46043-w)
Supplement: Supplementary file 1 — Supplementary Information. [file 41598_2023_46043_MOESM1_ESM.zip › Supplementary_files/Supplementary_all_revised.docx]

**Supplementary table S1.** Number of DMR per cluster in the T30-T0 comparison: statistics per window (cluster dimension) with 100 bp pace are reported. Yellow lines represent the chosen window.

| Window base pair | Average bp per DMR | DMR with 2 DMP | DMR with 3 DMP | DMR with 4 DMP | DMR /window |
| --- | --- | --- | --- | --- | --- |
| cluster_100 | 46 | 3 |  |  | 0,030 |
| cluster_200 | 142 | 26 |  |  | 0,130 |
| cluster_300 | 177 | 41 |  |  | 0,137 |
| cluster_400 | 229 | 58 |  |  | 0,145 |
| cluster_500 | 290 | 80 |  |  | 0,160 |
| cluster_600 | 357 | 99 | 2 |  | 0,168 |
| cluster_700 | 424 | 122 | 4 |  | 0,180 |
| cluster_800 | 456 | 133 | 3 | 1 | 0,171 |
| cluster_900 | 504 | 147 | 4 | 1 | 0,169 |
| cluster_1000 | 552 | 165 | 4 | 1 | 0,170 |
| cluster_1100 | 597 | 182 | 4 | 1 | 0,170 |
| cluster_1200 | 630 | 194 | 4 | 1 | 0,166 |
| cluster_1300 | 666 | 202 | 5 | 1 | 0,160 |
| cluster_1400 | 727 | 216 | 7 | 1 | 0,160 |
| cluster_1500 | 795 | 236 | 8 | 1 | 0,163 |
| cluster_1600 | 832 | 249 | 8 | 1 | 0,161 |
| cluster_1700 | 863 | 259 | 8 | 1 | 0,158 |
| cluster_1800 | 894 | 269 | 8 | 1 | 0,154 |
| cluster_1900 | 950 | 283 | 9 | 1 | 0,154 |
| cluster_2000 | 971 | 286 | 9 | 1 | 0,148 |

**Supplementary table S2.** Number of DMR per cluster in the T90-T0 comparison: statistics per window (cluster dimension) with 100 bp pace are reported. Yellow lines represent the chosen window.

| Window base pair | Average bp per DMR | DMR with 2 DMP | DMR with 3 DMP | DMR with 4 DMP | DMR /window |
| --- | --- | --- | --- | --- | --- |
| cluster_100 | 61 | 14 |  |  | 0,140 |
| cluster_200 | 144 | 78 | 1 |  | 0,395 |
| cluster_300 | 188 | 134 | 1 |  | 0,450 |
| cluster_400 | 230 | 182 | 2 |  | 0,460 |
| cluster_500 | 280 | 234 | 2 |  | 0,472 |
| cluster_600 | 340 | 299 | 3 |  | 0,503 |
| cluster_700 | 408 | 367 | 7 |  | 0,534 |
| cluster_800 | 453 | 405 | 10 | 1 | 0,520 |
| cluster_900 | 514 | 470 | 12 | 1 | 0,537 |
| cluster_1000 | 584 | 531 | 20 | 1 | 0,552 |
| cluster_1100 | 646 | 600 | 21 | 3 | 0,567 |
| cluster_1200 | 692 | 653 | 24 | 3 | 0,567 |
| cluster_1300 | 745 | 705 | 27 | 4 | 0,566 |
| cluster_1400 | 796 | 749 | 33 | 4 | 0,561 |
| cluster_1500 | 845 | 801 | 36 | 4 | 0,561 |
| cluster_1600 | 883 | 836 | 40 | 4 | 0,550 |
| cluster_1700 | 937 | 884 | 44 | 5 | 0,549 |
| cluster_1800 | 977 | 911 | 46 | 7 | 0,536 |
| cluster_1900 | 1034 | 965 | 50 | 7 | 0,538 |
| cluster_2000 | 1077 | 996 | 51 | 7 | 0,528 |

**Supplementary table S3.** Number of DMR per cluster in the T30-T90 comparison: statistics per window (cluster dimension) with 100 bp pace are reported. Yellow lines represent the chosen window.

| Window base pair | Average bp per DMR | DMR with 2 DMP | DMR with 3 DMP | DMR with 4 DMP | DMR /window |
| --- | --- | --- | --- | --- | --- |
| cluster_100 | 59 | 7 |  |  | 0,070 |
| cluster_200 | 147 | 39 |  |  | 0,195 |
| cluster_300 | 191 | 71 |  |  | 0,237 |
| cluster_400 | 223 | 87 | 1 |  | 0,220 |
| cluster_500 | 264 | 108 | 1 |  | 0,218 |
| cluster_600 | 327 | 139 | 1 |  | 0,233 |
| cluster_700 | 385 | 168 | 1 |  | 0,241 |
| cluster_800 | 434 | 183 | 4 |  | 0,234 |
| cluster_900 | 506 | 215 | 5 |  | 0,244 |
| cluster_1000 | 580 | 252 | 7 |  | 0,259 |
| cluster_1100 | 642 | 275 | 10 | 1 | 0,260 |
| cluster_1200 | 684 | 300 | 10 | 1 | 0,259 |
| cluster_1300 | 752 | 342 | 10 | 1 | 0,272 |
| cluster_1400 | 801 | 356 | 15 | 1 | 0,266 |
| cluster_1500 | 855 | 382 | 16 | 1 | 0,266 |
| cluster_1600 | 901 | 403 | 18 | 1 | 0,264 |
| cluster_1700 | 961 | 434 | 20 | 1 | 0,268 |
| cluster_1800 | 1007 | 453 | 21 | 2 | 0,264 |
| cluster_1900 | 1051 | 479 | 21 | 2 | 0,264 |
| cluster_2000 | 1086 | 495 | 22 | 2 | 0,260 |

**Supplementary table S4.** Differentially methylated genes for the three comparisons (light yellow bock for T30 *vs* T0, green for T90 *vs* T0 and light blue for T90 *vs* T30). In the last column, the DMR site relative to the gene portion is reported: “Regulation site (TSS)” is the transcription start site (2.5Kb upstream) of strand + genes, while the “Regulation site (TTS)” is the transcription termination site (2.5Kb downstream) of the strand - genes.

| Comparison | Gene_ID | Gene_Name | Methylation | Gene methylation site |
| --- | --- | --- | --- | --- |
| T30 vs T0 | ENSECAG00000008186 | *SP100* | -34.55 | Body |
|  | ENSECAG00000022473 | *MAST1* | -25.45 | Regulatory region (TSS) |
|  | ENSECAG00000008259 | *MASP1* | -23.75 | Body |
|  | ENSECAG00000021049 | *GDAP1L1* | -21.82 | Body |
|  | ENSECAG00000032719 |  | -21.25 | Body |
|  | ENSECAG00000010943 |  | -15.94 | Regulatory region (TSS) |
|  | ENSECAG00000023084 | *PRKCQ* | -12.94 | Body |
|  | ENSECAG00000020446 | *SLC7A4* | -12.83 | Body |
|  | ENSECAG00000020278 | *RAP1GAP2* | -12.08 | Body |
|  | ENSECAG00000000420 | *BANP* | -11.56 | Body |
|  | ENSECAG00000021312 | *ABHD14A* | 12.27 | Regulatory region (TSS) |
|  | ENSECAG00000022421 | *ABHD14B* | 12.27 | Regulatory region (TSS) |
|  | ENSECAG00000023902 | *BAK1* | 13.85 | Regulatory region (TSS) |
|  | ENSECAG00000004978 |  | 13.85 | Body |
|  | ENSECAG00000010628 | *PPP1R14B* | 14.07 | Regulatory region (TSS) |
|  | ENSECAG00000019602 | *DLEC1* | 14.15 | Body |
|  | ENSECAG00000018306 | *CREB3L1* | 14.40 | Body |
|  | ENSECAG00000009505 |  | 14.44 | Body |
|  | ENSECAG00000020246 | *SEPTIN9* | 15.29 | Body |
|  | ENSECAG00000020314 | *ANO1* | 15.56 | Body |
|  | ENSECAG00000000614 | *SNX27* | 15.56 | Body |
|  | ENSECAG00000006963 | *NACC2* | 16.84 | Body |
|  | ENSECAG00000038763 |  | 18.57 | Regulatory region (TTS) |
|  | ENSECAG00000000199 | *RGS3* | 18.67 | Body |
|  | ENSECAG00000016581 | *HRH2* | 18.89 | Body |
|  | ENSECAG00000011631 | *EPHB2* | 20.00 | Body |
|  | ENSECAG00000017937 | *FLVCR2* | 20.00 | Body |
|  | ENSECAG00000008644 | *MEAK7* | 20.00 | Body |
|  | ENSECAG00000018677 | *SCTR* | 20.00 | Body |
|  | ENSECAG00000022697 | *TRABD2B* | 20.00 | Body |
|  | ENSECAG00000039437 |  | 21.05 | Body |
|  | ENSECAG00000011150 | *TBC1D22A* | 21.25 | Body |
|  | ENSECAG00000018719 | *GRK7* | 21.67 | Body |
|  | ENSECAG00000022930 | *C7orf50* | 22.22 | Body |
|  | ENSECAG00000021212 | *CD209* | 22.22 | Body |
|  | ENSECAG00000022511 | *PDLIM7* | 23.16 | Regulatory region (TTS) |
|  | ENSECAG00000022261 | *FABP6* | 23.33 | Regulatory region (TSS) |
|  | ENSECAG00000026889 | *PPFIA1* | 23.33 | Body |
|  | ENSECAG00000021303 | *PWWP2A* | 23.33 | Body |
|  | ENSECAG00000011180 | *CD6* | 23.64 | Body |
|  | ENSECAG00000005816 | *CDHR2* | 23.64 | Body |
|  | ENSECAG00000036087 | *FBN3* | 23.64 | Body |
|  | ENSECAG00000003581 | *ZNF449* | 24.00 | Body |
|  | ENSECAG00000006447 |  | 24.00 | Regulatory region (TSS) |
|  | ENSECAG00000013604 |  | 25.00 | Regulatory region (TTS) |
|  | ENSECAG00000022844 |  | 25.00 | Body |
|  | ENSECAG00000012083 | *ATP2A3* | 25.19 | Body |
|  | ENSECAG00000001688 | *PCSK6* | 25.26 | Body |
|  | ENSECAG00000019083 | *SORL1* | 25.26 | Body |
|  | ENSECAG00000009891 | *ARMC5* | 25.45 | Regulatory region (TTS) |
|  | ENSECAG00000001963 | *TGFB1I1* | 25.45 | Regulatory region (TSS) |
|  | ENSECAG00000041368 |  | 25.45 | Body |
|  | ENSECAG00000016022 | *MADD* | 25.60 | Body |
|  | ENSECAG00000007808 | *DCBLD1* | 25.83 | Regulatory region (TSS) |
|  | ENSECAG00000037308 |  | 26.00 | Regulatory region (TSS) |
|  | ENSECAG00000002944 | *GRK2* | 26.67 | Body |
|  | ENSECAG00000018844 | *TUBGCP2* | 26.88 | Regulatory region (TTS) |
|  | ENSECAG00000028085 |  | 26.88 | Regulatory region (TSS) |
|  | ENSECAG00000024970 | *KCNQ1* | 27.04 | Body |
|  | ENSECAG00000026889 |  | 27.69 | Body |
|  | ENSECAG00000032084 |  | 28.00 | Body |
|  | ENSECAG00000024970 |  | 28.33 | Body |
|  | ENSECAG00000013543 | *TSPAN15* | 28.57 | Body |
|  | ENSECAG00000007317 | *NBL1* | 28.97 | Regulatory region (TTS) |
|  | ENSECAG00000026984 | *GPSM3* | 29.23 | Body |
|  | ENSECAG00000012302 | *NOTCH4* | 29.23 | Regulatory region (TTS) |
|  | ENSECAG00000003887 | *PBX2* | 29.23 | Regulatory region (TSS) |
|  | ENSECAG00000018169 | *RREB1* | 30.59 | Body |
|  | ENSECAG00000021392 | *COL26A1* | 30.91 | Body |
|  | ENSECAG00000041789 |  | 30.91 | Regulatory region (TTS) |
|  | ENSECAG00000020424 | *HDAC7* | 31.43 | Body |
|  | ENSECAG00000020427 | *DLGAP2* | 32.00 | Body |
|  | ENSECAG00000001597 | *LHX1* | 32.00 | Body |
|  | ENSECAG00000015951 | *PPIP5K1* | 32.31 | Regulatory region (TSS) |
|  | ENSECAG00000029964 |  | 32.73 | Body |
|  | ENSECAG00000041175 |  | 32.73 | Regulatory region (TTS) |
|  | ENSECAG00000020272 | *CPNE7* | 33.33 | Regulatory region (TSS) |
|  | ENSECAG00000018645 | *SPG7* | 33.33 | Body |
|  | ENSECAG00000002074 |  | 34.00 | Body |
|  | ENSECAG00000012853 | *SP6* | 34.55 | Regulatory region (TTS) |
|  | ENSECAG00000025011 | *C11orf86* | 36.00 | Regulatory region (TSS) |
|  | ENSECAG00000021446 | *GRID1* | 36.00 | Body |
|  | ENSECAG00000007534 | *RGS19* | 36.00 | Regulatory region (TTS) |
|  | ENSECAG00000020977 | *TCEA2* | 36.00 | Body |
|  | ENSECAG00000016850 | *KRIT1* | 36.67 | Body |
|  | ENSECAG00000012027 | *CACNA1E* | 42.67 | Body |
|  | ENSECAG00000041009 |  | 44.00 | Regulatory region (TTS) |
|  | ENSECAG00000023190 | *MICAL2* | 44.29 | Body |
| T90 vs T0 | ENSECAG00000009258 | *DAAM1* | -58.00 | Body |
|  | ENSECAG00000001342 | *SCAND1* | -56.36 | Body |
|  | ENSECAG00000043190 |  | -56.19 | Regulatory region (TSS) |
|  | ENSECAG00000043398 |  | -56.19 | Regulatory region (TTS) |
|  | ENSECAG00000012936 | *DSCAM* | -53.68 | Body |
|  | ENSECAG00000018782 | *BAIAP2L2* | -53.33 | Body |
|  | ENSECAG00000017871 | *RBMS3* | -51.76 | Regulatory region (TSS) |
|  | ENSECAG00000023756 | *SMYD4* | -50.98 | Regulatory region (TSS) |
|  | ENSECAG00000023886 | *RPA1* | -50.98 | Body |
|  | ENSECAG00000008976 | *EFCAB14* | -50.53 | Body |
|  | ENSECAG00000016897 | *ECSIT* | -50.00 | Body |
|  | ENSECAG00000014815 | *FXYD1* | -49.17 | Regulatory region (TTS) |
|  | ENSECAG00000028572 | *FXYD7* | -49.17 | Body |
|  | ENSECAG00000006022 | *TMEM104* | -48.89 | Body |
|  | ENSECAG00000021446 | *GRID1* | -48.75 | Body |
|  | ENSECAG00000008186 | *SP100* | -47.27 | Body |
|  | ENSECAG00000024715 | *FIBCD1* | -47.27 | Regulatory region (TSS) |
|  | ENSECAG00000010287 | *DPF1* | -47.14 | Body |
|  | ENSECAG00000023084 | *PRKCQ* | -47.06 | Body |
|  | ENSECAG00000006979 | *GALNT10* | -46.96 | Body |
|  | ENSECAG00000022834 | *WDR59* | -46.45 | Body |
|  | ENSECAG00000029712 |  | -46.23 | Regulatory region (TTS) |
|  | ENSECAG00000031874 |  | -46.23 | Body |
|  | ENSECAG00000032084 |  | -46.00 | Body |
|  | ENSECAG00000023646 | *EVI5* | -44.76 | Body |
|  | ENSECAG00000012751 | *CCKBR* | -44.00 | Body |
|  | ENSECAG00000020464 | *AJAP1* | -43.75 | Body |
|  | ENSECAG00000000482 | *BCL7C* | -43.64 | Body |
|  | ENSECAG00000024739 | *CSTPP1* | -43.64 | Body |
|  | ENSECAG00000024744 | *ARFGAP2* | -43.64 | Regulatory region (TTS) |
|  | ENSECAG00000000420 | *BANP* | -43.56 | Body |
|  | ENSECAG00000011631 | *EPHB2* | -43.33 | Body |
|  | ENSECAG00000012796 | *ABCB9* | -43.00 | Regulatory region (TTS) |
|  | ENSECAG00000015997 | *ESRRG* | -42.44 | Body |
|  | ENSECAG00000023388 | *ARPIN* | -42.35 | Body |
|  | ENSECAG00000015133 | *AP5Z1* | -42.22 | Regulatory region (TTS) |
|  | ENSECAG00000019718 | *RADIL* | -42.22 | Regulatory region (TTS) |
|  | ENSECAG00000037025 |  | -41.82 | Body |
|  | ENSECAG00000031172 | *ID1* | -41.43 | Regulatory region (TTS) |
|  | ENSECAG00000019400 | *KCNIP1* | -41.18 | Body |
|  | ENSECAG00000013321 | *NAPG* | -40.38 | Regulatory region (TTS) |
|  | ENSECAG00000017555 | *TF* | -40.00 | Body |
|  | ENSECAG00000024948 | *SLCO2A1* | -40.00 | Regulatory region (TTS) |
|  | ENSECAG00000035568 |  | -40.00 | Body |
|  | ENSECAG00000016197 | *NWD1* | -38.46 | Body |
|  | ENSECAG00000032711 | *POLRMT* | -38.46 | Body |
|  | ENSECAG00000013807 | *IQSEC3* | -38.26 | Body |
|  | ENSECAG00000006829 | *KAZN* | -38.18 | Body |
|  | ENSECAG00000026889 | *PPFIA1* | -37.69 | Body |
|  | ENSECAG00000035894 | *FAM53B* | -37.14 | Body |
|  | ENSECAG00000030369 |  | -37.04 | Body |
|  | ENSECAG00000013935 | *MTUS2* | -36.47 | Body |
|  | ENSECAG00000013542 | *CMIP* | -36.36 | Body |
|  | ENSECAG00000022473 | *MAST1* | -36.36 | Regulatory region (TSS) |
|  | ENSECAG00000023320 |  | -36.25 | Body |
|  | ENSECAG00000014486 |  | -36.00 | Body |
|  | ENSECAG00000021037 | *FEZF2* | -36.00 | Regulatory region (TTS) |
|  | ENSECAG00000000686 | *LAPTM5* | -35.71 | Body |
|  | ENSECAG00000011697 | *TMCC3* | -35.00 | Body |
|  | ENSECAG00000016662 | *MCF2L* | -35.00 | Body |
|  | ENSECAG00000021049 | *GDAP1L1* | -34.55 | Body |
|  | ENSECAG00000022504 | *CTBP2* | -34.29 | Body |
|  | ENSECAG00000040680 |  | -34.12 | Body |
|  | ENSECAG00000000918 | *RORC* | -34.00 | Body |
|  | ENSECAG00000011939 | *CLIP2* | -34.00 | Body |
|  | ENSECAG00000023922 | *E2F2* | -34.00 | Body |
|  | ENSECAG00000005905 | *SMOX* | -33.33 | Body |
|  | ENSECAG00000006829 | *KAZN* | -33.33 | Body |
|  | ENSECAG00000023512 | *MTA3* | -32.50 | Body |
|  | ENSECAG00000016645 | *MCOLN1* | -31.67 | Body |
|  | ENSECAG00000034713 |  | -31.67 | Regulatory region (TSS) |
|  | ENSECAG00000035156 | *ZNF358* | -31.67 | Regulatory region (TTS) |
|  | ENSECAG00000038405 |  | -31.67 | Regulatory region (TTS) |
|  | ENSECAG00000006686 | *NCAPG2* | -31.03 | Body |
|  | ENSECAG00000034732 | *PCDHB13* | -30.91 | Body |
|  | ENSECAG00000000657 | *CDCP2* | -30.77 | Body |
|  | ENSECAG00000039607 |  | -30.64 | Body |
|  | ENSECAG00000022258 | *SDSL* | -30.37 | Body |
|  | ENSECAG00000008246 | *GRIP1* | -30.34 | Body |
|  | ENSECAG00000012483 | *TPRG1L* | -30.00 | Body |
|  | ENSECAG00000000282 | *ABCD4* | -29.23 | Body |
|  | ENSECAG00000020995 | *NLGN3* | -28.80 | Body |
|  | ENSECAG00000018464 | *SLC35G4* | -28.48 | Body |
|  | ENSECAG00000007682 | *STK24* | -28.33 | Body |
|  | ENSECAG00000002520 | *SLC45A4* | -28.18 | Body |
|  | ENSECAG00000021955 |  | -28.00 | Body |
|  | ENSECAG00000020526 | *KCNQ2* | -27.69 | Body |
|  | ENSECAG00000000922 | *ASIC2* | -27.50 | Body |
|  | ENSECAG00000022378 | *IGSF3* | -27.50 | Body |
|  | ENSECAG00000023130 | *AP1M2* | -27.50 | Body |
|  | ENSECAG00000013322 | *PNKD* | -27.27 | Body |
|  | ENSECAG00000017385 | *ZC3HAV1L* | -27.27 | Body |
|  | ENSECAG00000024770 | *HSPBAP1* | -27.06 | Body |
|  | ENSECAG00000030487 | *ITPK1* | -27.06 | Body |
|  | ENSECAG00000023889 | *SYT13* | -26.67 | Regulatory region (TSS) |
|  | ENSECAG00000022257 | *NRGN* | -26.15 | Body |
|  | ENSECAG00000012555 | *ZDHHC12* | -26.00 | Body |
|  | ENSECAG00000010267 | *CD44* | -25.88 | Body |
|  | ENSECAG00000035102 |  | -25.83 | Body |
|  | ENSECAG00000024970 | *KCNQ1* | -25.45 | Body |
|  | ENSECAG00000022127 | *PLAUR* | -25.00 | Body |
|  | ENSECAG00000020446 | *SLC7A4* | -24.78 | Body |
|  | ENSECAG00000007104 | *NISCH* | -24.62 | Body |
|  | ENSECAG00000032834 |  | -24.62 | Body |
|  | ENSECAG00000026942 | *RABL2B* | -24.44 | Body |
|  | ENSECAG00000014473 | *SEC14L1* | -24.29 | Body |
|  | ENSECAG00000019949 | *CYP4F8* | -24.00 | Body |
|  | ENSECAG00000032059 |  | -24.00 | Body |
|  | ENSECAG00000009213 | *WNT7A* | -23.64 | Body |
|  | ENSECAG00000005015 | *ARHGEF10L* | -23.33 | Body |
|  | ENSECAG00000013593 | *OSR1* | -23.08 | Body |
|  | ENSECAG00000016537 | *IL12RB2* | -22.86 | Body |
|  | ENSECAG00000022366 | *TLE6* | -21.82 | Regulatory region (TSS) |
|  | ENSECAG00000020583 | *SMAD7* | -21.67 | Body |
|  | ENSECAG00000026889 | *PPFIA1* | -21.25 | Body |
|  | ENSECAG00000016824 | *NRP2* | -21.18 | Body |
|  | ENSECAG00000003656 | *NANOS1* | -20.00 | Regulatory region (TSS) |
|  | ENSECAG00000007750 | *DDX39B* | -20.00 | Body |
|  | ENSECAG00000009140 | *KLHL25* | -20.00 | Body |
|  | ENSECAG00000020232 | *EDAR* | -20.00 | Body |
|  | ENSECAG00000031878 | *IGLV8-61* | -20.00 | Body |
|  | ENSECAG00000035532 | *HLA-E* | -20.00 | Body |
|  | ENSECAG00000043378 |  | -20.00 | Body |
|  | ENSECAG00000007657 | *SFSWAP* | -19.31 | Regulatory region (TTS) |
|  | ENSECAG00000015344 | *RYR1* | -18.75 | Body |
|  | ENSECAG00000023303 | *ARHGAP22* | -18.54 | Body |
|  | ENSECAG00000013951 | *PPP2R2C* | -18.33 | Body |
|  | ENSECAG00000018470 | *CCDC60* | -18.33 | Body |
|  | ENSECAG00000023613 | *IGSF21* | -18.18 | Body |
|  | ENSECAG00000016246 | *ASAP1* | -18.10 | Body |
|  | ENSECAG00000019504 | *SYN3* | -18.00 | Body |
|  | ENSECAG00000016565 | *EEFSEC* | -17.69 | Body |
|  | ENSECAG00000016976 | *RARB* | -17.65 | Regulatory region (TSS) |
|  | ENSECAG00000006886 | *RAB31* | -17.50 | Body |
|  | ENSECAG00000026838 | *C1orf87* | -17.33 | Regulatory region (TSS) |
|  | ENSECAG00000005622 |  | -17.14 | Regulatory region (TSS) |
|  | ENSECAG00000034493 |  | -16.92 | Body |
|  | ENSECAG00000031575 | *HOXC4* | -16.25 | Regulatory region (TSS) |
|  | ENSECAG00000038539 | *UBALD2* | -14.74 | Body |
|  | ENSECAG00000024970 | *KCNQ1* | -13.33 | Body |
|  | ENSECAG00000008566 | *CTSE* | -9.41 | Body |
|  | ENSECAG00000033397 | *CACNG8* | 11.33 | Body |
|  | ENSECAG00000040004 | *CD300H* | 12.00 | Regulatory region (TSS) |
|  | ENSECAG00000043506 | *CD300C* | 12.00 | Body |
|  | ENSECAG00000022511 | *PDLIM7* | 12.11 | Regulatory region (TTS) |
|  | ENSECAG00000009505 |  | 12.22 | Body |
|  | ENSECAG00000029177 |  | 12.80 | Regulatory region (TTS) |
|  | ENSECAG00000040382 |  | 12.80 | Regulatory region (TTS) |
|  | ENSECAG00000009524 | *SLC16A3* | 12.94 | Body |
|  | ENSECAG00000034848 |  | 12.94 | Regulatory region (TTS) |
|  | ENSECAG00000003668 | *CBFA2T3* | 13.00 | Body |
|  | ENSECAG00000010403 | *BARHL2* | 13.04 | Body |
|  | ENSECAG00000037736 |  | 13.91 | Regulatory region (TSS) |
|  | ENSECAG00000020534 | *DCST2* | 14.17 | Regulatory region (TTS) |
|  | ENSECAG00000038153 | *ZBTB7B* | 14.17 | Body |
|  | ENSECAG00000024574 | *GATA3* | 14.44 | Body |
|  | ENSECAG00000037272 | *VSTM2L* | 14.67 | Body |
|  | ENSECAG00000032719 |  | 15.00 | Body |
|  | ENSECAG00000037030 |  | 15.00 | Body |
|  | ENSECAG00000009793 | *TAGLN3* | 15.20 | Body |
|  | ENSECAG00000019507 | *SOGA1* | 15.29 | Body |
|  | ENSECAG00000023776 | *TTK* | 15.56 | Body |
|  | ENSECAG00000021142 | *NKAP* | 15.83 | Body |
|  | ENSECAG00000032785 |  | 16.00 | Body |
|  | ENSECAG00000020216 | *KRT7* | 16.36 | Body |
|  | ENSECAG00000039058 | *KRT6C* | 16.36 | Body |
|  | ENSECAG00000031244 |  | 16.47 | Body |
|  | ENSECAG00000001688 | *PCSK6* | 16.84 | Body |
|  | ENSECAG00000015408 | *PCYOX1* | 16.92 | Body |
|  | ENSECAG00000013487 |  | 17.30 | Body |
|  | ENSECAG00000020042 | *GAS8* | 17.30 | Regulatory region (TTS) |
|  | ENSECAG00000015234 | *JAKMIP3* | 17.33 | Body |
|  | ENSECAG00000016022 | *MADD* | 17.60 | Body |
|  | ENSECAG00000020551 | *AHCY* | 17.65 | Body |
|  | ENSECAG00000022125 | *TNNT2* | 17.69 | Body |
|  | ENSECAG00000014975 | *PXN* | 17.78 | Body |
|  | ENSECAG00000011631 | *EPHB2* | 17.86 | Regulatory region (TSS) |
|  | ENSECAG00000005816 | *CDHR2* | 18.18 | Body |
|  | ENSECAG00000015508 | *IPO13* | 18.33 | Body |
|  | ENSECAG00000018681 | *RIPOR1* | 18.33 | Body |
|  | ENSECAG00000042942 |  | 18.33 | Body |
|  | ENSECAG00000000951 | *SCUBE1* | 18.57 | Body |
|  | ENSECAG00000020234 | *TUBB2B* | 18.57 | Body |
|  | ENSECAG00000036329 |  | 18.89 | Regulatory region (TTS) |
|  | ENSECAG00000037803 |  | 19.35 | Body |
|  | ENSECAG00000014550 | *AP2A2* | 19.47 | Body |
|  | ENSECAG00000000199 | *RGS3* | 20.00 | Body |
|  | ENSECAG00000000665 | *TBC1D17* | 20.00 | Regulatory region (TTS) |
|  | ENSECAG00000005957 | *FMNL3* | 20.00 | Body |
|  | ENSECAG00000009865 | *NFAM1* | 20.00 | Body |
|  | ENSECAG00000010499 | *TRABD2A* | 20.00 | Body |
|  | ENSECAG00000010584 | *DYNC1LI1* | 20.00 | Body |
|  | ENSECAG00000012311 | *SEC14L5* | 20.00 | Regulatory region (TSS) |
|  | ENSECAG00000012813 | *TSEN2* | 20.00 | Body |
|  | ENSECAG00000013171 | *RNF216* | 20.00 | Body |
|  | ENSECAG00000018105 | *IL4I1* | 20.00 | Body |
|  | ENSECAG00000020626 | *SDK2* | 20.00 | Body |
|  | ENSECAG00000020939 | *RNF215* | 20.00 | Body |
|  | ENSECAG00000022930 | *C7orf50* | 20.74 | Body |
|  | ENSECAG00000015466 | *LRRC20* | 21.18 | Body |
|  | ENSECAG00000042538 | *PCDHB5* | 21.18 | Body |
|  | ENSECAG00000019096 | *CACNA1C* | 21.25 | Body |
|  | ENSECAG00000010099 | *SLC26A11* | 21.29 | Body |
|  | ENSECAG00000012498 | *CD5* | 21.29 | Regulatory region (TTS) |
|  | ENSECAG00000012866 | *VPS37C* | 21.29 | Regulatory region (TTS) |
|  | ENSECAG00000008644 | *MEAK7* | 21.33 | Body |
|  | ENSECAG00000032084 |  | 21.33 | Body |
|  | ENSECAG00000008108 | *PLEKHM3* | 21.43 | Body |
|  | ENSECAG00000000638 | *SEL1L3* | 21.54 | Body |
|  | ENSECAG00000022989 | *FEZF1* | 21.54 | Body |
|  | ENSECAG00000003388 | *RNF220* | 21.82 | Body |
|  | ENSECAG00000007127 | *HS3ST2* | 21.82 | Regulatory region (TSS) |
|  | ENSECAG00000007455 | *CARD14* | 21.82 | Body |
|  | ENSECAG00000018844 | *TUBGCP2* | 21.88 | Regulatory region (TTS) |
|  | ENSECAG00000028085 |  | 21.88 | Regulatory region (TSS) |
|  | ENSECAG00000008684 | *HTT* | 22.00 | Body |
|  | ENSECAG00000042489 |  | 22.00 | Regulatory region (TSS) |
|  | ENSECAG00000006963 | *NACC2* | 22.11 | Body |
|  | ENSECAG00000009122 |  | 22.14 | Body |
|  | ENSECAG00000013671 | *EIF3B* | 22.14 | Body |
|  | ENSECAG00000008974 | *FOXP1* | 22.22 | Body |
|  | ENSECAG00000000637 | *STAC2* | 22.35 | Body |
|  | ENSECAG00000016306 | *GRM4* | 22.35 | Body |
|  | ENSECAG00000025080 | *RPL19* | 22.35 | Regulatory region (TSS) |
|  | ENSECAG00000025086 | *DNMT3A* | 22.35 | Body |
|  | ENSECAG00000042273 | *CACNB1* | 22.35 | Body |
|  | ENSECAG00000009598 | *OLIG3* | 22.50 | Regulatory region (TTS) |
|  | ENSECAG00000015962 | *MUCL3* | 22.50 | Body |
|  | ENSECAG00000022844 |  | 22.50 | Body |
|  | ENSECAG00000012921 | *ALPK2* | 22.67 | Body |
|  | ENSECAG00000023190 | *MICAL2* | 22.86 | Body |
|  | ENSECAG00000024957 | *SHFL* | 23.08 | Regulatory region (TSS) |
|  | ENSECAG00000033356 | *ZNF787* | 23.08 | Body |
|  | ENSECAG00000033531 |  | 23.16 | Body |
|  | ENSECAG00000009210 | *STK32C* | 23.33 | Regulatory region (TTS) |
|  | ENSECAG00000017084 | *DPYSL4* | 23.33 | Body |
|  | ENSECAG00000022174 | *KLHDC1* | 23.33 | Body |
|  | ENSECAG00000031215 | *TEX22* | 23.33 | Body |
|  | ENSECAG00000019897 | *DDX56* | 23.43 | Body |
|  | ENSECAG00000007647 |  | 23.64 | Body |
|  | ENSECAG00000008645 | *PORCN* | 23.64 | Regulatory region (TTS) |
|  | ENSECAG00000022178 | *EBP* | 23.64 | Body |
|  | ENSECAG00000000199 |  | 24.00 | Body |
|  | ENSECAG00000001597 | *LHX1* | 24.00 | Body |
|  | ENSECAG00000009387 | *MEIS1* | 24.00 | Body |
|  | ENSECAG00000010591 | *LTK* | 24.00 | Regulatory region (TTS) |
|  | ENSECAG00000013661 | *FAM184B* | 24.00 | Body |
|  | ENSECAG00000018981 | *ITPKA* | 24.00 | Body |
|  | ENSECAG00000020246 | *SEPTIN9* | 24.00 | Body |
|  | ENSECAG00000020955 | *SIGLEC6* | 24.00 | Body |
|  | ENSECAG00000038698 | *ALOX15* | 24.00 | Body |
|  | ENSECAG00000008392 | *SLC8A2* | 24.14 | Body |
|  | ENSECAG00000018392 | *KPTN* | 24.14 | Body |
|  | ENSECAG00000007808 | *DCBLD1* | 24.17 | Regulatory region (TSS) |
|  | ENSECAG00000016679 | *GALNT15* | 24.21 | Body |
|  | ENSECAG00000002049 |  | 24.29 | Body |
|  | ENSECAG00000009265 | *QPCTL* | 24.38 | Regulatory region (TTS) |
|  | ENSECAG00000032958 | *FBXO46* | 24.38 | Regulatory region (TTS) |
|  | ENSECAG00000013142 | *AAGAB* | 24.62 | Body |
|  | ENSECAG00000017045 | *PRDM16* | 24.62 | Body |
|  | ENSECAG00000019504 | *SYN3* | 24.76 | Body |
|  | ENSECAG00000018306 | *CREB3L1* | 24.80 | Body |
|  | ENSECAG00000023681 | *AQP2* | 24.80 | Body |
|  | ENSECAG00000003260 | *ITIH5* | 25.00 | Body |
|  | ENSECAG00000013604 |  | 25.00 | Regulatory region (TTS) |
|  | ENSECAG00000033945 |  | 25.00 | Body |
|  | ENSECAG00000038896 | *MRPL24* | 25.00 | Body |
|  | ENSECAG00000036087 | *FBN3* | 25.45 | Body |
|  | ENSECAG00000041368 |  | 25.45 | Body |
|  | ENSECAG00000008592 | *GLT6D1* | 25.56 | Body |
|  | ENSECAG00000014994 | *G6PD* | 26.00 | Regulatory region (TTS) |
|  | ENSECAG00000025088 | *PLXNA3* | 26.00 | Body |
|  | ENSECAG00000032591 |  | 26.00 | Body |
|  | ENSECAG00000023780 | *VSX2* | 26.15 | Body |
|  | ENSECAG00000039477 |  | 26.15 | Body |
|  | ENSECAG00000043634 |  | 26.15 | Regulatory region (TTS) |
|  | ENSECAG00000008381 | *NR2F2* | 26.67 | Body |
|  | ENSECAG00000013196 | *ENO4* | 26.67 | Body |
|  | ENSECAG00000016231 | *HSPA12A* | 26.67 | Body |
|  | ENSECAG00000019235 | *TOLLIP* | 26.67 | Body |
|  | ENSECAG00000020427 | *DLGAP2* | 26.67 | Body |
|  | ENSECAG00000021212 | *CD209* | 26.67 | Body |
|  | ENSECAG00000024970 | *KCNQ1* | 26.67 | Body |
|  | ENSECAG00000037313 |  | 26.67 | Regulatory region (TSS) |
|  | ENSECAG00000041041 |  | 26.67 | Body |
|  | ENSECAG00000019559 | *WHRN* | 26.90 | Body |
|  | ENSECAG00000022504 | *CTBP2* | 26.92 | Body |
|  | ENSECAG00000005729 | *RRAGA* | 27.06 | Body |
|  | ENSECAG00000013176 | *MEGF6* | 27.06 | Body |
|  | ENSECAG00000015621 | *ARHGEF16* | 27.06 | Body |
|  | ENSECAG00000009598 | *OLIG3* | 27.14 | Regulatory region (TTS) |
|  | ENSECAG00000013543 | *TSPAN15* | 27.14 | Body |
|  | ENSECAG00000023779 | *ARMCX1* | 27.14 | Regulatory region (TSS) |
|  | ENSECAG00000020314 | *ANO1* | 27.22 | Body |
|  | ENSECAG00000007080 | *ALK* | 27.27 | Body |
|  | ENSECAG00000021392 | *COL26A1* | 27.27 | Body |
|  | ENSECAG00000010136 | *PPP3CA* | 27.37 | Body |
|  | ENSECAG00000017805 | *HSD17B4* | 27.37 | Body |
|  | ENSECAG00000021051 | *FLI1* | 27.37 | Body |
|  | ENSECAG00000037030 |  | 27.54 | Body |
|  | ENSECAG00000000201 | *SOHLH2* | 27.62 | Body |
|  | ENSECAG00000016371 | *CACNA1A* | 27.69 | Body |
|  | ENSECAG00000029378 |  | 27.83 | Regulatory region (TTS) |
|  | ENSECAG00000018142 | *CYB561A3* | 27.86 | Body |
|  | ENSECAG00000021331 | *NOTCH3* | 28.00 | Body |
|  | ENSECAG00000011180 | *CD6* | 28.18 | Body |
|  | ENSECAG00000016706 | *ENC1* | 28.33 | Regulatory region (TSS) |
|  | ENSECAG00000024970 | *KCNQ1* | 28.33 | Body |
|  | ENSECAG00000026889 | *PPFIA1* | 28.33 | Body |
|  | ENSECAG00000020207 | *PAX7* | 28.57 | Body |
|  | ENSECAG00000036896 | *ASCL1* | 28.57 | Body |
|  | ENSECAG00000037992 |  | 28.57 | Body |
|  | ENSECAG00000007134 | *AP2A1* | 28.70 | Regulatory region (TTS) |
|  | ENSECAG00000019538 | *FUZ* | 28.70 | Body |
|  | ENSECAG00000029326 |  | 28.70 | Regulatory region (TSS) |
|  | ENSECAG00000024970 | *KCNQ1* | 28.89 | Body |
|  | ENSECAG00000017518 | *COL25A1* | 29.00 | Regulatory region (TSS) |
|  | ENSECAG00000017859 | *FKBP6* | 29.00 | Regulatory region (TTS) |
|  | ENSECAG00000022880 | *HIP1* | 29.00 | Body |
|  | ENSECAG00000017467 | *KCNQ3* | 29.09 | Body |
|  | ENSECAG00000019286 | *TTC24* | 29.09 | Body |
|  | ENSECAG00000020111 | *IQGAP3* | 29.09 | Body |
|  | ENSECAG00000021312 | *ABHD14A* | 29.09 | Regulatory region (TSS) |
|  | ENSECAG00000022421 | *ABHD14B* | 29.09 | Regulatory region (TSS) |
|  | ENSECAG00000029626 |  | 29.09 | Body |
|  | ENSECAG00000013347 | *CCN6* | 29.23 | Body |
|  | ENSECAG00000016337 | *GALNT6* | 29.23 | Body |
|  | ENSECAG00000015058 | *ARHGEF3* | 29.33 | Body |
|  | ENSECAG00000019690 | *NFIX* | 29.52 | Body |
|  | ENSECAG00000038235 |  | 29.52 | Body |
|  | ENSECAG00000039487 | *SOX7* | 29.57 | Regulatory region (TSS) |
|  | ENSECAG00000012550 | *SUSD4* | 29.60 | Body |
|  | ENSECAG00000033903 |  | 29.62 | Regulatory region (TSS) |
|  | ENSECAG00000035312 |  | 29.62 | Body |
|  | ENSECAG00000035860 | *RNA5S9* | 29.62 | Regulatory region (TTS) |
|  | ENSECAG00000001603 | *PEG10* | 30.00 | Body |
|  | ENSECAG00000002830 | *STRN4* | 30.00 | Body |
|  | ENSECAG00000014150 | *PTK2* | 30.00 | Body |
|  | ENSECAG00000020155 | *SGCE* | 30.00 | Regulatory region (TSS) |
|  | ENSECAG00000020891 | *SLC29A3* | 30.00 | Body |
|  | ENSECAG00000021446 | *GRID1* | 30.00 | Body |
|  | ENSECAG00000042841 |  | 30.00 | Body |
|  | ENSECAG00000012083 | *ATP2A3* | 30.37 | Body |
|  | ENSECAG00000010104 | *KCNIP2* | 30.50 | Body |
|  | ENSECAG00000020347 | *ARMH3* | 30.50 | Regulatory region (TTS) |
|  | ENSECAG00000013054 | *TFDP1* | 30.59 | Regulatory region (TTS) |
|  | ENSECAG00000020199 | *ATP4B* | 30.59 | Regulatory region (TTS) |
|  | ENSECAG00000032346 |  | 30.67 | Body |
|  | ENSECAG00000038763 |  | 30.71 | Regulatory region (TTS) |
|  | ENSECAG00000009328 | *LRIG1* | 30.83 | Body |
|  | ENSECAG00000012853 | *SP6* | 30.91 | Regulatory region (TTS) |
|  | ENSECAG00000030409 |  | 30.91 | Body |
|  | ENSECAG00000031932 | *TMEM63C* | 30.91 | Body |
|  | ENSECAG00000038271 |  | 30.91 | Regulatory region (TSS) |
|  | ENSECAG00000041107 |  | 30.91 | Regulatory region (TTS) |
|  | ENSECAG00000043553 |  | 31.00 | Body |
|  | ENSECAG00000015710 | *FGF5* | 31.25 | Body |
|  | ENSECAG00000020424 | *HDAC7* | 31.43 | Body |
|  | ENSECAG00000009996 | *YBX2* | 31.58 | Body |
|  | ENSECAG00000015095 | *NCF1* | 31.67 | Body |
|  | ENSECAG00000010378 | *CCNY* | 31.76 | Body |
|  | ENSECAG00000018169 | *RREB1* | 31.76 | Body |
|  | ENSECAG00000008485 | *ITGAL* | 32.00 | Regulatory region (TSS) |
|  | ENSECAG00000019602 | *DLEC1* | 32.20 | Body |
|  | ENSECAG00000019716 | *CCDC88C* | 32.28 | Body |
|  | ENSECAG00000000113 | *ACOT11* | 32.31 | Body |
|  | ENSECAG00000003887 | *PBX2* | 32.31 | Regulatory region (TSS) |
|  | ENSECAG00000012302 | *NOTCH4* | 32.31 | Regulatory region (TTS) |
|  | ENSECAG00000026984 | *GPSM3* | 32.31 | Body |
|  | ENSECAG00000028779 |  | 32.31 | Regulatory region (TTS) |
|  | ENSECAG00000032121 |  | 32.63 | Body |
|  | ENSECAG00000010087 | *GNAI3* | 32.67 | Body |
|  | ENSECAG00000016589 | *RFX1* | 32.73 | Body |
|  | ENSECAG00000013322 | *PNKD* | 32.80 | Body |
|  | ENSECAG00000012166 | *LRP1* | 32.86 | Body |
|  | ENSECAG00000015574 | *NFIA* | 32.86 | Body |
|  | ENSECAG00000021729 | *SRRM4* | 33.00 | Body |
|  | ENSECAG00000020682 | *TTLL11* | 33.33 | Body |
|  | ENSECAG00000021016 | *SLC41A3* | 33.55 | Body |
|  | ENSECAG00000024970 | *KCNQ1* | 33.60 | Body |
|  | ENSECAG00000014491 | *NLRC5* | 33.68 | Body |
|  | ENSECAG00000010480 | *EIF5AL1* | 33.85 | Body |
|  | ENSECAG00000034037 |  | 33.85 | Regulatory region (TSS) |
|  | ENSECAG00000006305 | *TTC21A* | 34.00 | Body |
|  | ENSECAG00000011334 | *SALL1* | 34.00 | Regulatory region (TSS) |
|  | ENSECAG00000014550 | *AP2A2* | 34.00 | Body |
|  | ENSECAG00000023717 | *IQSEC1* | 34.00 | Body |
|  | ENSECAG00000021672 | *PSAP* | 34.12 | Regulatory region (TSS) |
|  | ENSECAG00000010078 | *CIT* | 34.29 | Body |
|  | ENSECAG00000022374 | *TBX6* | 34.29 | Regulatory region (TSS) |
|  | ENSECAG00000024038 | *YPEL3* | 34.29 | Body |
|  | ENSECAG00000017160 | *TBXAS1* | 34.40 | Body |
|  | ENSECAG00000007317 | *NBL1* | 34.48 | Regulatory region (TTS) |
|  | ENSECAG00000002222 | *ZBTB6* | 35.00 | Regulatory region (TSS) |
|  | ENSECAG00000012342 | *CRIPT* | 35.33 | Body |
|  | ENSECAG00000012487 | *PIGF* | 35.33 | Regulatory region (TSS) |
|  | ENSECAG00000022103 | *SBF1* | 35.71 | Body |
|  | ENSECAG00000032622 | *HMX1* | 35.79 | Regulatory region (TSS) |
|  | ENSECAG00000039437 |  | 35.79 | Body |
|  | ENSECAG00000002074 |  | 36.00 | Body |
|  | ENSECAG00000008630 | *TOR1B* | 36.36 | Body |
|  | ENSECAG00000008984 | *CROCC* | 36.47 | Body |
|  | ENSECAG00000023518 | *TP53TG5* | 36.47 | Regulatory region (TTS) |
|  | ENSECAG00000021552 | *RBM20* | 36.67 | Body |
|  | ENSECAG00000014210 | *CCM2L* | 36.77 | Body |
|  | ENSECAG00000018591 | *CTU1* | 37.14 | Regulatory region (TTS) |
|  | ENSECAG00000038373 |  | 37.14 | Body |
|  | ENSECAG00000022697 | *TRABD2B* | 37.50 | Body |
|  | ENSECAG00000017725 | *BCAN* | 38.18 | Body |
|  | ENSECAG00000019173 | *FAM13A* | 38.18 | Body |
|  | ENSECAG00000031195 |  | 38.18 | Body |
|  | ENSECAG00000031671 | *TUBA3E* | 38.18 | Body |
|  | ENSECAG00000012264 | *TMCC2* | 38.33 | Body |
|  | ENSECAG00000014699 | *NUAK2* | 38.33 | Body |
|  | ENSECAG00000000718 | *TAF15* | 38.75 | Body |
|  | ENSECAG00000033909 |  | 38.95 | Body |
|  | ENSECAG00000036896 | *ASCL1* | 39.00 | Body |
|  | ENSECAG00000011907 | *C19orf54* | 39.17 | Regulatory region (TSS) |
|  | ENSECAG00000013141 | *SNRPA* | 39.17 | Body |
|  | ENSECAG00000008343 | *PAX4* | 39.38 | Regulatory region (TSS) |
|  | ENSECAG00000004978 |  | 39.49 | Body |
|  | ENSECAG00000023902 | *BAK1* | 39.49 | Regulatory region (TSS) |
|  | ENSECAG00000002988 | *PSTPIP1* | 40.00 | Body |
|  | ENSECAG00000018719 | *GRK7* | 40.00 | Body |
|  | ENSECAG00000022068 | *RCN1* | 40.00 | Body |
|  | ENSECAG00000041789 |  | 40.00 | Regulatory region (TTS) |
|  | ENSECAG00000019400 | *KCNIP1* | 40.69 | Body |
|  | ENSECAG00000000614 | *SNX27* | 40.74 | Body |
|  | ENSECAG00000010919 | *MYO9B* | 40.87 | Body |
|  | ENSECAG00000009714 | *RIN3* | 41.54 | Body |
|  | ENSECAG00000009213 | *WNT7A* | 42.00 | Body |
|  | ENSECAG00000016572 | *UPK3BL2* | 42.11 | Regulatory region (TTS) |
|  | ENSECAG00000036553 | *TLR5* | 42.31 | Regulatory region (TSS) |
|  | ENSECAG00000021303 | *PWWP2A* | 43.33 | Body |
|  | ENSECAG00000022261 | *FABP6* | 43.33 | Regulatory region (TSS) |
|  | ENSECAG00000021013 | *SHISAL2A* | 44.00 | Regulatory region (TTS) |
|  | ENSECAG00000002944 | *GRK2* | 44.44 | Body |
|  | ENSECAG00000011167 | *ESRRB* | 44.73 | Body |
|  | ENSECAG00000017728 | *CAPN12* | 45.45 | Body |
|  | ENSECAG00000014577 | *ADAM28* | 45.56 | Body |
|  | ENSECAG00000026889 | *PPFIA1* | 45.56 | Body |
|  | ENSECAG00000016399 | *AQR* | 45.71 | Regulatory region (TSS) |
|  | ENSECAG00000015951 | *PPIP5K1* | 46.15 | Regulatory region (TSS) |
|  | ENSECAG00000036280 |  | 48.75 | Body |
|  | ENSECAG00000023663 | *LRP4* | 50.91 | Body |
|  | ENSECAG00000016123 | *PCDHGA4* | 61.67 | Body |
| T90 vs T30 | ENSECAG00000018622 | *TTC7A* | -61.00 | Body |
|  | ENSECAG00000015133 | *AP5Z1* | -54.44 | Regulatory region (TTS) |
|  | ENSECAG00000019718 | *RADIL* | -54.44 | Regulatory region (TTS) |
|  | ENSECAG00000021392 | *COL26A1* | -48.42 | Body |
|  | ENSECAG00000001342 | *SCAND1* | -48.18 | Body |
|  | ENSECAG00000016169 | *COL6A6* | -46.00 | Body |
|  | ENSECAG00000023646 | *EVI5* | -45.71 | Body |
|  | ENSECAG00000006979 | *GALNT10* | -44.35 | Body |
|  | ENSECAG00000024739 | *CSTPP1* | -43.64 | Body |
|  | ENSECAG00000024744 | *ARFGAP2* | -43.64 | Regulatory region (TTS) |
|  | ENSECAG00000031172 | *ID1* | -42.86 | Regulatory region (TTS) |
|  | ENSECAG00000008976 | *EFCAB14* | -42.11 | Body |
|  | ENSECAG00000000482 | *BCL7C* | -41.82 | Body |
|  | ENSECAG00000037025 |  | -40.00 | Body |
|  | ENSECAG00000012796 | *ABCB9* | -39.00 | Regulatory region (TTS) |
|  | ENSECAG00000022834 | *WDR59* | -37.42 | Body |
|  | ENSECAG00000026889 | *PPFIA1* | -37.27 | Body |
|  | ENSECAG00000000291 | *PLCG2* | -37.14 | Body |
|  | ENSECAG00000014815 | *FXYD1* | -36.67 | Regulatory region (TTS) |
|  | ENSECAG00000028572 | *FXYD7* | -36.67 | Body |
|  | ENSECAG00000035102 |  | -36.67 | Body |
|  | ENSECAG00000043434 |  | -36.67 | Body |
|  | ENSECAG00000029964 |  | -36.36 | Body |
|  | ENSECAG00000041175 |  | -36.36 | Regulatory region (TTS) |
|  | ENSECAG00000015938 | *SSRP1* | -36.15 | Regulatory region (TSS) |
|  | ENSECAG00000016640 | *P2RX3* | -36.15 | Regulatory region (TSS) |
|  | ENSECAG00000009258 | *DAAM1* | -36.00 | Body |
|  | ENSECAG00000011939 | *CLIP2* | -36.00 | Body |
|  | ENSECAG00000029588 | *GNG2* | -35.71 | Body |
|  | ENSECAG00000023756 | *SMYD4* | -35.61 | Regulatory region (TSS) |
|  | ENSECAG00000023886 | *RPA1* | -35.61 | Body |
|  | ENSECAG00000019504 | *SYN3* | -34.39 | Body |
|  | ENSECAG00000010287 | *DPF1* | -34.29 | Body |
|  | ENSECAG00000022504 | *CTBP2* | -34.29 | Body |
|  | ENSECAG00000023084 | *PRKCQ* | -34.12 | Body |
|  | ENSECAG00000002520 | *SLC45A4* | -33.64 | Body |
|  | ENSECAG00000043190 |  | -33.33 | Regulatory region (TSS) |
|  | ENSECAG00000043398 |  | -33.33 | Regulatory region (TTS) |
|  | ENSECAG00000023512 | *MTA3* | -32.50 | Body |
|  | ENSECAG00000000622 | *CDK18* | -31.85 | Body |
|  | ENSECAG00000023320 |  | -31.25 | Body |
|  | ENSECAG00000019236 | *LDLRAD3* | -31.03 | Body |
|  | ENSECAG00000022257 | *NRGN* | -30.77 | Body |
|  | ENSECAG00000020464 | *AJAP1* | -30.00 | Body |
|  | ENSECAG00000026887 | *TSPAN8* | -30.00 | Regulatory region (TTS) |
|  | ENSECAG00000032084 |  | -30.00 | Body |
|  | ENSECAG00000038405 |  | -30.00 | Regulatory region (TTS) |
|  | ENSECAG00000013321 | *NAPG* | -29.06 | Regulatory region (TTS) |
|  | ENSECAG00000005655 |  | -28.89 | Regulatory region (TTS) |
|  | ENSECAG00000029924 |  | -28.89 | Body |
|  | ENSECAG00000006873 | *FOXI1* | -28.57 | Body |
|  | ENSECAG00000032609 |  | -28.57 | Regulatory region (TTS) |
|  | ENSECAG00000035894 | *FAM53B* | -28.57 | Body |
|  | ENSECAG00000026889 | *PPFIA1* | -28.46 | Body |
|  | ENSECAG00000007682 | *STK24* | -28.33 | Body |
|  | ENSECAG00000040744 | *TYROBP* | -28.28 | Regulatory region (TSS) |
|  | ENSECAG00000000657 | *CDCP2* | -27.69 | Body |
|  | ENSECAG00000032834 |  | -27.69 | Body |
|  | ENSECAG00000033206 | *VN1R5* | -27.50 | Body |
|  | ENSECAG00000040368 |  | -27.50 | Body |
|  | ENSECAG00000031341 | *SURF4* | -27.41 | Body |
|  | ENSECAG00000034732 | *PCDHB13* | -27.27 | Body |
|  | ENSECAG00000017522 | *TNFSF14* | -26.67 | Body |
|  | ENSECAG00000012751 | *CCKBR* | -26.40 | Body |
|  | ENSECAG00000020995 | *NLGN3* | -26.40 | Body |
|  | ENSECAG00000010400 | *CLN5* | -26.15 | Body |
|  | ENSECAG00000006519 | *SMG6* | -26.00 | Body |
|  | ENSECAG00000031878 | *IGLV8-61* | -25.45 | Body |
|  | ENSECAG00000026889 | *PPFIA1* | -25.38 | Body |
|  | ENSECAG00000022127 | *PLAUR* | -25.00 | Body |
|  | ENSECAG00000023889 | *SYT13* | -25.00 | Regulatory region (TSS) |
|  | ENSECAG00000009935 | *PTK6* | -24.29 | Regulatory region (TSS) |
|  | ENSECAG00000010972 | *SRMS* | -24.29 | Regulatory region (TTS) |
|  | ENSECAG00000014486 |  | -24.00 | Body |
|  | ENSECAG00000021037 | *FEZF2* | -24.00 | Regulatory region (TTS) |
|  | ENSECAG00000035568 |  | -24.00 | Body |
|  | ENSECAG00000011697 | *TMCC3* | -23.75 | Body |
|  | ENSECAG00000020925 | *TMCO4* | -23.64 | Body |
|  | ENSECAG00000010267 | *CD44* | -23.53 | Body |
|  | ENSECAG00000022301 | *PDXK* | -23.23 | Body |
|  | ENSECAG00000006686 | *NCAPG2* | -22.76 | Body |
|  | ENSECAG00000030487 | *ITPK1* | -22.35 | Body |
|  | ENSECAG00000033042 |  | -22.35 | Regulatory region (TTS) |
|  | ENSECAG00000011120 | *THRA* | -22.11 | Body |
|  | ENSECAG00000011944 | *SNRPB* | -22.00 | Body |
|  | ENSECAG00000016897 | *ECSIT* | -22.00 | Body |
|  | ENSECAG00000040452 |  | -22.00 | Regulatory region (TTS) |
|  | ENSECAG00000008397 | *RFX8* | -21.82 | Body |
|  | ENSECAG00000017864 | *FMOD* | -21.82 | Body |
|  | ENSECAG00000033909 |  | -21.82 | Body |
|  | ENSECAG00000013593 | *OSR1* | -21.54 | Body |
|  | ENSECAG00000010628 | *PPP1R14B* | -21.48 | Regulatory region (TSS) |
|  | ENSECAG00000014031 | *B4GALNT3* | -21.00 | Body |
|  | ENSECAG00000005015 | *ARHGEF10L* | -20.83 | Body |
|  | ENSECAG00000000686 | *LAPTM5* | -20.00 | Body |
|  | ENSECAG00000005292 | *PCMT1* | -20.00 | Body |
|  | ENSECAG00000007534 | *RGS19* | -20.00 | Regulatory region (TTS) |
|  | ENSECAG00000011934 | *LGR6* | -20.00 | Body |
|  | ENSECAG00000013935 | *MTUS2* | -20.00 | Body |
|  | ENSECAG00000014473 | *SEC14L1* | -20.00 | Body |
|  | ENSECAG00000020977 | *TCEA2* | -20.00 | Body |
|  | ENSECAG00000035240 | *RAET1E* | -20.00 | Regulatory region (TSS) |
|  | ENSECAG00000036563 | *RAET1L* | -20.00 | Body |
|  | ENSECAG00000031575 | *HOXC4* | -18.75 | Regulatory region (TSS) |
|  | ENSECAG00000009273 | *SLC45A3* | -18.67 | Regulatory region (TSS) |
|  | ENSECAG00000022258 | *SDSL* | -18.52 | Body |
|  | ENSECAG00000023130 | *AP1M2* | -18.33 | Body |
|  | ENSECAG00000017830 | *TBC1D9B* | -18.18 | Body |
|  | ENSECAG00000023289 | *CCDC27* | -18.10 | Body |
|  | ENSECAG00000007602 | *LTV1* | -17.78 | Body |
|  | ENSECAG00000028923 |  | -17.78 | Regulatory region (TTS) |
|  | ENSECAG00000039607 |  | -17.66 | Body |
|  | ENSECAG00000016791 | *MEIS3* | -17.65 | Regulatory region (TSS) |
|  | ENSECAG00000016645 | *MCOLN1* | -16.67 | Body |
|  | ENSECAG00000034713 |  | -16.67 | Regulatory region (TSS) |
|  | ENSECAG00000035156 | *ZNF358* | -16.67 | Regulatory region (TTS) |
|  | ENSECAG00000009505 |  | -15.56 | Body |
|  | ENSECAG00000042883 | *SPTBN4* | -15.56 | Body |
|  | ENSECAG00000016246 | *ASAP1* | -15.24 | Body |
|  | ENSECAG00000022378 | *IGSF3* | -15.00 | Body |
|  | ENSECAG00000020526 | *KCNQ2* | -14.62 | Body |
|  | ENSECAG00000016565 | *EEFSEC* | -13.08 | Body |
|  | ENSECAG00000022208 | *SHANK2* | -13.08 | Body |
|  | ENSECAG00000011700 | *PLCB3* | -12.97 | Regulatory region (TTS) |
|  | ENSECAG00000014950 | *BAD* | -12.97 | Regulatory region (TTS) |
|  | ENSECAG00000007657 | *SFSWAP* | -12.41 | Regulatory region (TTS) |
|  | ENSECAG00000020446 | *SLC7A4* | -11.96 | Body |
|  | ENSECAG00000020721 | *PPM1L* | -10.91 | Body |
|  | ENSECAG00000007104 | *NISCH* | -10.77 | Body |
|  | ENSECAG00000020278 | *RAP1GAP2* | 8.28 | Body |
|  | ENSECAG00000037853 |  | 9.27 | Body |
|  | ENSECAG00000037030 |  | 10.49 | Body |
|  | ENSECAG00000020814 | *CLEC16A* | 12.67 | Body |
|  | ENSECAG00000028292 | *SRXN1* | 12.86 | Regulatory region (TTS) |
|  | ENSECAG00000015641 | *DSCC1* | 13.22 | Body |
|  | ENSECAG00000020634 | *NT5M* | 13.33 | Body |
|  | ENSECAG00000024272 | *RIMS1* | 13.68 | Body |
|  | ENSECAG00000000199 | *RGS3* | 13.91 | Body |
|  | ENSECAG00000024970 | *KCNQ1* | 14.40 | Body |
|  | ENSECAG00000033397 | *CACNG8* | 14.67 | Body |
|  | ENSECAG00000019559 | *WHRN* | 15.17 | Body |
|  | ENSECAG00000008649 | *CCDC12* | 15.65 | Body |
|  | ENSECAG00000032213 |  | 15.65 | Body |
|  | ENSECAG00000019400 | *KCNIP1* | 15.86 | Body |
|  | ENSECAG00000037918 |  | 16.00 | Regulatory region (TSS) |
|  | ENSECAG00000038592 |  | 16.00 | Regulatory region (TTS) |
|  | ENSECAG00000041032 |  | 16.00 | Body |
|  | ENSECAG00000015710 | *FGF5* | 16.25 | Body |
|  | ENSECAG00000020557 | *KIAA1522* | 16.43 | Body |
|  | ENSECAG00000021672 | *PSAP* | 16.47 | Regulatory region (TSS) |
|  | ENSECAG00000019104 | *REN* | 17.04 | Body |
|  | ENSECAG00000031195 |  | 17.27 | Body |
|  | ENSECAG00000031671 | *TUBA3E* | 17.27 | Body |
|  | ENSECAG00000012680 | *N4BP2L1* | 17.50 | Body |
|  | ENSECAG00000022132 | *EPHA8* | 17.60 | Body |
|  | ENSECAG00000002944 | *GRK2* | 17.78 | Body |
|  | ENSECAG00000023321 | *ELF5* | 17.78 | Body |
|  | ENSECAG00000016371 | *CACNA1A* | 18.46 | Body |
|  | ENSECAG00000022504 | *CTBP2* | 18.46 | Body |
|  | ENSECAG00000033636 |  | 18.46 | Body |
|  | ENSECAG00000008645 | *PORCN* | 18.57 | Regulatory region (TTS) |
|  | ENSECAG00000022178 | *EBP* | 18.57 | Body |
|  | ENSECAG00000014210 | *CCM2L* | 18.71 | Body |
|  | ENSECAG00000021016 | *SLC41A3* | 18.71 | Body |
|  | ENSECAG00000008984 | *CROCC* | 18.82 | Body |
|  | ENSECAG00000032121 |  | 18.95 | Body |
|  | ENSECAG00000007080 | *ALK* | 20.00 | Body |
|  | ENSECAG00000014577 | *ADAM28* | 20.00 | Body |
|  | ENSECAG00000036329 |  | 20.00 | Regulatory region (TTS) |
|  | ENSECAG00000024952 | *TEKT1* | 21.33 | Body |
|  | ENSECAG00000005752 | *PACS2* | 21.71 | Body |
|  | ENSECAG00000013081 | *MMP9* | 21.82 | Regulatory region (TTS) |
|  | ENSECAG00000024422 | *CDH22* | 21.82 | Body |
|  | ENSECAG00000011524 | *CAP2* | 22.61 | Body |
|  | ENSECAG00000005015 | *ARHGEF10L* | 22.67 | Body |
|  | ENSECAG00000010087 | *GNAI3* | 22.67 | Body |
|  | ENSECAG00000002049 |  | 22.86 | Body |
|  | ENSECAG00000009071 | *LSM12* | 23.08 | Regulatory region (TSS) |
|  | ENSECAG00000024363 | *G6PC3* | 23.08 | Regulatory region (TSS) |
|  | ENSECAG00000009793 | *TAGLN3* | 23.20 | Body |
|  | ENSECAG00000024242 | *SCARB1* | 23.33 | Body |
|  | ENSECAG00000010901 | *CTNND2* | 23.64 | Body |
|  | ENSECAG00000018151 | *OPTC* | 24.00 | Regulatory region (TSS) |
|  | ENSECAG00000014150 | *PTK2* | 24.44 | Body |
|  | ENSECAG00000022251 | *MAP3K15* | 24.71 | Body |
|  | ENSECAG00000029177 |  | 24.80 | Regulatory region (TTS) |
|  | ENSECAG00000040382 |  | 24.80 | Regulatory region (TTS) |
|  | ENSECAG00000021729 | *SRRM4* | 25.00 | Body |
|  | ENSECAG00000013487 |  | 25.41 | Body |
|  | ENSECAG00000020042 | *GAS8* | 25.41 | Regulatory region (TTS) |
|  | ENSECAG00000009865 | *NFAM1* | 25.45 | Body |
|  | ENSECAG00000021665 | *SGMS2* | 25.45 | Body |
|  | ENSECAG00000000296 | *LAMC3* | 25.83 | Body |
|  | ENSECAG00000038468 |  | 25.83 | Regulatory region (TSS) |
|  | ENSECAG00000041500 |  | 26.00 | Body |
|  | ENSECAG00000024991 | *ARPC1B* | 26.11 | Body |
|  | ENSECAG00000023776 | *TTK* | 26.67 | Body |
|  | ENSECAG00000002988 | *PSTPIP1* | 27.27 | Body |
|  | ENSECAG00000027754 | *CDX2* | 27.78 | Regulatory region (TSS) |
|  | ENSECAG00000009387 | *MEIS1* | 28.00 | Body |
|  | ENSECAG00000012921 | *ALPK2* | 28.00 | Body |
|  | ENSECAG00000021331 | *NOTCH3* | 28.00 | Body |
|  | ENSECAG00000015408 | *PCYOX1* | 28.46 | Body |
|  | ENSECAG00000016337 | *GALNT6* | 29.23 | Body |
|  | ENSECAG00000006771 | *PRKCA* | 30.00 | Body |
|  | ENSECAG00000014550 | *AP2A2* | 30.00 | Body |
|  | ENSECAG00000010104 | *KCNIP2* | 30.50 | Body |
|  | ENSECAG00000020347 | *ARMH3* | 30.50 | Regulatory region (TTS) |
|  | ENSECAG00000022559 | *FIBP* | 30.63 | Regulatory region (TSS) |
|  | ENSECAG00000031061 | *CCDC85B* | 30.63 | Regulatory region (TSS) |
|  | ENSECAG00000009095 | *RNF44* | 30.77 | Body |
|  | ENSECAG00000036484 |  | 30.77 | Body |
|  | ENSECAG00000041727 |  | 31.43 | Regulatory region (TTS) |
|  | ENSECAG00000037030 |  | 33.13 | Body |
|  | ENSECAG00000021031 | *PLCG1* | 35.29 | Body |
|  | ENSECAG00000042538 | *PCDHB5* | 35.29 | Body |
|  | ENSECAG00000019772 | *PTPN1* | 36.00 | Body |
|  | ENSECAG00000032719 |  | 36.25 | Body |
|  | ENSECAG00000016862 | *SLC25A33* | 36.36 | Body |
|  | ENSECAG00000038761 | *ABHD17A* | 36.67 | Body |
|  | ENSECAG00000015058 | *ARHGEF3* | 40.00 | Body |
|  | ENSECAG00000012813 | *TSEN2* | 48.00 | Body |
|  | ENSECAG00000036280 |  | 50.00 | Body |
|  | ENSECAG00000018490 | *HIVEP3* | 52.86 | Body |
|  | ENSECAG00000016399 | *AQR* | 58.57 | Regulatory region (TSS) |
|  | ENSECAG00000008974 | *FOXP1* | 59.26 | Body |
|  | ENSECAG00000019813 | *NFATC1* | 64.62 | Body |
|  | ENSECAG00000020035 | *SLC52A3* | 72.86 | Regulatory region (TSS) |

**Supplementary table S6.** Check for selected genes (10% most de-methylated and methylated for each comparison) of expression levels horse PBMCs and human PBMCs. The levels of methylation for selected genes are reported as well as the mean transcript per million (TPM) of the five horses at rest (basal) and after the gallop race (post race) and human cells. Colored blocks represent the comparison where genes resulted differentially methylated: light yellow bock for T30 *vs* T0, green for T90 *vs* T0, light blue for T90 *vs* T30, yellow for *Early response* and blue for *Mid response*.

| Comparison | Horse Ensembl gene ID | Human Ensembl gene ID | Gene name | Methylation | Horse TPM basal  PBMCs | Horse TPM post race  PBMCs | Human TPM  PBMCs |
| --- | --- | --- | --- | --- | --- | --- | --- |
| T30 vs T0 | ENSECAG00000008186 | ENSG00000067066 | SP100 | -34.5 | 22.2 | 25.6 | 12.5 |
|  | ENSECAG00000022473 | ENSG00000105613 | MAST1 | -25.5 | 4.8 | 5.1 | 0.6 |
|  | ENSECAG00000008259 |  | MASP1 | -23.8 | 0.0 | 0.0 |  |
|  | ENSECAG00000021049 |  | GDAP1L1 | -21.8 | 0.0 | 0.0 |  |
|  | ENSECAG00000032719 |  |  | -21.3 |  |  |  |
|  | ENSECAG00000010943 |  |  | -15.9 | 0.0 | 0.0 |  |
|  | ENSECAG00000023084 | ENSG00000065675 | PRKCQ | -12.9 | 3.6 | 3.5 | 20.5 |
|  | ENSECAG00000020446 |  | SLC7A4 | -12.8 | 0.1 | 0.1 |  |
|  | ENSECAG00000012853 | ENSG00000189120 | SP6 | 34.5 | 0.0 | 0.0 | 0.1 |
|  | ENSECAG00000007534 |  | RGS19 | 36.0 | 103.3 | 72.9 |  |
|  | ENSECAG00000020977 | ENSG00000171703 | TCEA2 | 36.0 | 17.2 | 19.2 | 2.5 |
|  | ENSECAG00000021446 |  | GRID1 | 36.0 | 0.0 | 0.0 |  |
|  | ENSECAG00000025011 |  | C11orf86 | 36.0 | 0.0 | 0.0 |  |
|  | ENSECAG00000016850 | ENSG00000001631 | KRIT1 | 36.7 | 4.7 | 3.8 | 11.8 |
|  | ENSECAG00000012027 |  | CACNA1E | 42.7 | 0.0 | 0.0 |  |
|  | ENSECAG00000041009 |  |  | 44.0 |  |  |  |
|  | ENSECAG00000023190 | ENSG00000133816 | MICAL2 | 44.3 | 0.0 | 0.0 | 13.0 |
| T90 vs T0 | ENSECAG00000009258 | ENSG00000100592 | DAAM1 | -58.0 | 0.8 | 1.0 | 11.5 |
|  | ENSECAG00000001342 | ENSG00000171222 | SCAND1 | -56.4 | 352.2 | 308.4 | 97.8 |
|  | ENSECAG00000043190 |  |  | -56.2 |  |  |  |
|  | ENSECAG00000043398 |  |  | -56.2 |  |  |  |
|  | ENSECAG00000012936 |  | DSCAM | -53.7 | 0.0 | 0.0 |  |
|  | ENSECAG00000018782 | ENSG00000128298 | BAIAP2L2 | -53.3 | 0.0 | 0.0 | 0.5 |
|  | ENSECAG00000017871 | ENSG00000203506 | RBMS3 | -51.8 | 0.0 | 0.0 | 0.2 |
|  | ENSECAG00000023756 | ENSG00000186532 | SMYD4 | -51.0 | 1.4 | 1.3 | 3.5 |
|  | ENSECAG00000023886 | ENSG00000132383 | RPA1 | -51.0 | 0.0 | 0.0 | 83.5 |
|  | ENSECAG00000008976 | ENSG00000159658 | EFCAB14 | -50.5 | 6.8 | 5.6 | 25.5 |
|  | ENSECAG00000016897 | ENSG00000130159 | ECSIT | -50.0 | 11.4 | 8.5 | 44.8 |
|  | ENSECAG00000014815 | ENSG00000266964 | FXYD1 | -49.2 | 1.0 | 0.5 | 0.1 |
|  | ENSECAG00000028572 | ENSG00000221946 | FXYD7 | -49.2 |  |  | 0.2 |
|  | ENSECAG00000006022 | ENSG00000109066 | TMEM104 | -48.9 | 1.3 | 1.4 | 6.5 |
|  | ENSECAG00000021446 |  | GRID1 | -48.8 | 0.0 | 0.0 |  |
|  | ENSECAG00000008186 | ENSG00000067066 | SP100 | -47.3 | 22.2 | 25.6 | 12.5 |
|  | ENSECAG00000024715 |  | FIBCD1 | -47.3 | 0.0 | 0.0 |  |
|  | ENSECAG00000010287 | ENSG00000011332 | DPF1 | -47.1 | 0.0 | 0.0 | 0.4 |
|  | ENSECAG00000023084 | ENSG00000065675 | PRKCQ | -47.1 | 3.6 | 3.5 | 20.5 |
|  | ENSECAG00000006979 | ENSG00000164574 | GALNT10 | -47.0 | 1.0 | 1.1 | 42.3 |
|  | ENSECAG00000022834 | ENSG00000103091 | WDR59 | -46.5 | 3.4 | 3.0 | 14.8 |
|  | ENSECAG00000029712 |  |  | -46.2 |  |  |  |
|  | ENSECAG00000031874 |  |  | -46.2 |  |  |  |
|  | ENSECAG00000032084 |  |  | -46.0 |  |  |  |
|  | ENSECAG00000023646 | ENSG00000067208 | EVI5 | -44.8 | 0.2 | 0.2 | 3.8 |
|  | ENSECAG00000012751 |  | CCKBR | -44.0 | 0.0 | 0.0 |  |
|  | ENSECAG00000020464 |  | AJAP1 | -43.8 | 0.0 | 0.0 |  |
|  | ENSECAG00000000482 | ENSG00000099385 | BCL7C | -43.6 | 63.2 | 57.2 | 28.5 |
|  | ENSECAG00000024739 |  | CSTPP1 | -43.6 | 0.0 | 0.0 |  |
|  | ENSECAG00000024744 | ENSG00000149182 | ARFGAP2 | -43.6 | 68.3 | 51.9 | 41.5 |
|  | ENSECAG00000000420 | ENSG00000172530 | BANP | -43.6 | 0.0 | 0.0 | 2.8 |
|  | ENSECAG00000011631 | ENSG00000133216 | EPHB2 | -43.3 | 0.1 | 0.2 | 0.4 |
|  | ENSECAG00000012796 | ENSG00000150967 | ABCB9 | -43.0 | 0.3 | 0.2 | 1.0 |
|  | ENSECAG00000015997 |  | ESRRG | -42.4 | 0.0 | 0.0 |  |
|  | ENSECAG00000023388 | ENSG00000242498 | ARPIN | -42.4 | 6.1 | 5.2 | 0.6 |
|  | ENSECAG00000015133 | ENSG00000242802 | AP5Z1 | -42.2 | 2.8 | 2.6 | 10.5 |
|  | ENSECAG00000019718 | ENSG00000157927 | RADIL | -42.2 | 0.0 | 0.0 | 0.3 |
|  | ENSECAG00000037025 |  |  | -41.8 |  |  |  |
|  | ENSECAG00000031172 | ENSG00000125968 | ID1 | -41.4 |  |  | 14.0 |
|  | ENSECAG00000019400 |  | KCNIP1 | -41.2 | 0.0 | 0.0 |  |
|  | ENSECAG00000013321 | ENSG00000134265 | NAPG | -40.4 | 4.8 | 3.3 | 10.3 |
|  | ENSECAG00000017555 | ENSG00000210049 | TF | -40.0 | 4.0 | 2.6 | 16.5 |
|  | ENSECAG00000024948 |  | SLCO2A1 | -40.0 | 0.0 | 0.0 |  |
|  | ENSECAG00000035568 |  |  | -40.0 |  |  |  |
|  | ENSECAG00000008984 | ENSG00000058453 | CROCC | 36.5 | 2.9 | 2.7 | 1.8 |
|  | ENSECAG00000023518 | ENSG00000124251 | TP53TG5 | 36.5 | 0.3 | 0.3 | 0.1 |
|  | ENSECAG00000021552 | ENSG00000203867 | RBM20 | 36.7 | 0.0 | 0.0 | 0.1 |
|  | ENSECAG00000014210 | ENSG00000101331 | CCM2L | 36.8 | 0.4 | 0.1 | 0.1 |
|  | ENSECAG00000018591 | ENSG00000142544 | CTU1 | 37.1 | 5.1 | 2.9 | 13.0 |
|  | ENSECAG00000038373 |  |  | 37.1 |  |  |  |
|  | ENSECAG00000022697 |  | TRABD2B | 37.5 | 0.0 | 0.0 |  |
|  | ENSECAG00000017725 | ENSG00000132692 | BCAN | 38.2 | 0.0 | 0.0 | 0.1 |
|  | ENSECAG00000019173 | ENSG00000138640 | FAM13A | 38.2 | 0.0 | 0.0 | 6.0 |
|  | ENSECAG00000031195 |  |  | 38.2 |  |  |  |
|  | ENSECAG00000031671 | ENSG00000152086 | TUBA3E | 38.2 |  |  | 0.1 |
|  | ENSECAG00000012264 | ENSG00000133069 | TMCC2 | 38.3 | 0.7 | 0.7 | 23.3 |
|  | ENSECAG00000014699 | ENSG00000163545 | NUAK2 | 38.3 | 21.2 | 29.6 | 1.0 |
|  | ENSECAG00000000718 | ENSG00000270647 | TAF15 | 38.8 | 40.5 | 35.5 | 90.3 |
|  | ENSECAG00000033909 |  |  | 38.9 |  |  |  |
|  | ENSECAG00000036896 |  | ASCL1 | 39.0 |  |  |  |
|  | ENSECAG00000011907 | ENSG00000188493 | C19orf54 | 39.2 | 6.2 | 5.9 | 12.5 |
|  | ENSECAG00000013141 | ENSG00000077312 | SNRPA | 39.2 | 14.7 | 11.9 | 194.3 |
|  | ENSECAG00000008343 |  | PAX4 | 39.4 | 0.0 | 0.0 |  |
|  | ENSECAG00000004978 |  |  | 39.5 | 0.7 | 0.6 |  |
|  | ENSECAG00000023902 | ENSG00000030110 | BAK1 | 39.5 | 107.3 | 93.8 | 22.0 |
|  | ENSECAG00000002988 | ENSG00000140368 | PSTPIP1 | 40.0 | 0.1 | 0.1 | 1.1 |
|  | ENSECAG00000018719 |  | GRK7 | 40.0 | 0.0 | 0.0 |  |
|  | ENSECAG00000022068 | ENSG00000049449 | RCN1 | 40.0 | 0.8 | 0.4 | 14.5 |
|  | ENSECAG00000041789 |  |  | 40.0 |  |  |  |
|  | ENSECAG00000019400 |  | KCNIP1 | 40.7 |  |  |  |
|  | ENSECAG00000000614 | ENSG00000143376 | SNX27 | 40.7 | 4.1 | 2.7 | 4.0 |
|  | ENSECAG00000010919 | ENSG00000099331 | MYO9B | 40.9 | 5.1 | 5.7 | 8.5 |
|  | ENSECAG00000009714 | ENSG00000100599 | RIN3 | 41.5 | 1.8 | 1.7 | 13.5 |
|  | ENSECAG00000009213 | ENSG00000154764 | WNT7A | 42.0 | 0.0 | 0.0 | 0.2 |
|  | ENSECAG00000016572 |  | UPK3BL2 | 42.1 | 0.0 | 0.0 |  |
|  | ENSECAG00000036553 | ENSG00000187554 | TLR5 | 42.3 |  |  | 0.7 |
|  | ENSECAG00000021303 | ENSG00000170234 | PWWP2A | 43.3 | 0.0 | 0.0 | 7.0 |
|  | ENSECAG00000022261 | ENSG00000170231 | FABP6 | 43.3 | 0.0 | 0.0 | 0.3 |
|  | ENSECAG00000021013 | ENSG00000182183 | SHISAL2A | 44.0 | 0.0 | 0.1 | 0.3 |
|  | ENSECAG00000002944 | ENSG00000173020 | GRK2 | 44.4 | 217.0 | 180.8 | 26.5 |
|  | ENSECAG00000011167 | ENSG00000196482 | ESRRB | 44.7 | 0.0 | 0.0 | 0.1 |
|  | ENSECAG00000017728 | ENSG00000182472 | CAPN12 | 45.5 | 0.0 | 0.0 | 0.1 |
|  | ENSECAG00000014577 | ENSG00000042980 | ADAM28 | 45.6 | 0.8 | 0.5 | 0.6 |
|  | ENSECAG00000026889 | ENSG00000131626 | PPFIA1 | 45.6 | 4.0 | 3.7 | 6.3 |
|  | ENSECAG00000016399 | ENSG00000021776 | AQR | 45.7 | 4.8 | 4.6 | 11.8 |
|  | ENSECAG00000015951 | ENSG00000168781 | PPIP5K1 | 46.2 | 0.3 | 0.2 | 2.3 |
|  | ENSECAG00000036280 |  |  | 48.8 |  |  |  |
|  | ENSECAG00000023663 | ENSG00000134569 | LRP4 | 50.9 | 0.1 | 0.0 | 0.1 |
|  | ENSECAG00000016123 |  | PCDHGA4 | 61.7 | 0.1 | 0.2 |  |
| T90 vs T30 | ENSECAG00000018622 | ENSG00000068724 | TTC7A | -61.0 | 3.7 | 4.5 | 9.3 |
|  | ENSECAG00000015133 | ENSG00000242802 | AP5Z1 | -54.4 | 2.8 | 2.6 | 10.5 |
|  | ENSECAG00000019718 | ENSG00000157927 | RADIL | -54.4 | 0.0 | 0.0 | 0.3 |
|  | ENSECAG00000021392 | ENSG00000160963 | COL26A1 | -48.4 | 0.0 | 0.0 | 0.2 |
|  | ENSECAG00000001342 | ENSG00000171222 | SCAND1 | -48.2 | 352.2 | 308.4 | 97.8 |
|  | ENSECAG00000016169 |  | COL6A6 | -46.0 | 0.0 | 0.0 |  |
|  | ENSECAG00000023646 | ENSG00000067208 | EVI5 | -45.7 | 0.2 | 0.2 | 3.8 |
|  | ENSECAG00000006979 | ENSG00000164574 | GALNT10 | -44.3 | 1.0 | 1.1 | 42.3 |
|  | ENSECAG00000024739 |  | CSTPP1 | -43.6 | 0.0 | 0.0 |  |
|  | ENSECAG00000024744 | ENSG00000149182 | ARFGAP2 | -43.6 | 68.3 | 51.9 | 41.5 |
|  | ENSECAG00000031172 | ENSG00000125968 | ID1 | -42.9 |  |  | 14.0 |
|  | ENSECAG00000008976 | ENSG00000159658 | EFCAB14 | -42.1 | 6.8 | 5.6 | 25.5 |
|  | ENSECAG00000000482 | ENSG00000099385 | BCL7C | -41.8 | 63.2 | 57.2 | 28.5 |
|  | ENSECAG00000037025 |  |  | -40.0 |  |  |  |
|  | ENSECAG00000012796 | ENSG00000150967 | ABCB9 | -39.0 | 0.3 | 0.2 | 1.0 |
|  | ENSECAG00000022834 | ENSG00000103091 | WDR59 | -37.4 | 3.4 | 3.0 | 14.8 |
|  | ENSECAG00000026889 | ENSG00000131626 | PPFIA1 | -37.3 | 4.0 | 3.7 | 6.3 |
|  | ENSECAG00000000291 | ENSG00000197943 | PLCG2 | -37.1 | 8.7 | 8.6 | 1.8 |
|  | ENSECAG00000014815 | ENSG00000266964 | FXYD1 | -36.7 | 1.0 | 0.5 | 0.1 |
|  | ENSECAG00000028572 | ENSG00000221946 | FXYD7 | -36.7 |  |  | 0.2 |
|  | ENSECAG00000035102 |  |  | -36.7 |  |  |  |
|  | ENSECAG00000043434 |  |  | -36.7 |  |  |  |
|  | ENSECAG00000029964 |  |  | -36.4 |  |  |  |
|  | ENSECAG00000041175 |  |  | -36.4 |  |  |  |
|  | ENSECAG00000006771 | ENSG00000154229 | PRKCA | 30.0 | 0.2 | 0.2 | 1.5 |
|  | ENSECAG00000014550 | ENSG00000183020 | AP2A2 | 30.0 | 6.5 | 5.7 | 13.0 |
|  | ENSECAG00000010104 | ENSG00000120049 | KCNIP2 | 30.5 | 1.4 | 1.0 | 0.5 |
|  | ENSECAG00000020347 | ENSG00000120029 | ARMH3 | 30.5 | 0.6 | 0.5 | 13.0 |
|  | ENSECAG00000022559 | ENSG00000172500 | FIBP | 30.6 | 69.2 | 51.6 | 39.3 |
|  | ENSECAG00000031061 | ENSG00000175602 | CCDC85B | 30.6 |  |  | 100.0 |
|  | ENSECAG00000009095 | ENSG00000146083 | RNF44 | 30.8 | 122.5 | 121.1 | 11.0 |
|  | ENSECAG00000036484 |  |  | 30.8 |  |  |  |
|  | ENSECAG00000041727 |  |  | 31.4 |  |  |  |
|  | ENSECAG00000037030 |  |  | 33.1 |  |  |  |
|  | ENSECAG00000021031 | ENSG00000124181 | PLCG1 | 35.3 | 99.9 | 121.9 | 5.5 |
|  | ENSECAG00000042538 | ENSG00000113209 | PCDHB5 | 35.3 |  |  | 0.1 |
|  | ENSECAG00000019772 | ENSG00000196396 | PTPN1 | 36.0 | 14.2 | 19.0 | 21.8 |
|  | ENSECAG00000032719 |  |  | 36.3 |  |  |  |
|  | ENSECAG00000016862 | ENSG00000171612 | SLC25A33 | 36.4 | 2.9 | 4.7 | 9.0 |
|  | ENSECAG00000038761 | ENSG00000129968 | ABHD17A | 36.7 |  |  | 24.5 |
|  | ENSECAG00000015058 | ENSG00000163947 | ARHGEF3 | 40.0 | 7.7 | 13.5 | 3.8 |
|  | ENSECAG00000012813 | ENSG00000154743 | TSEN2 | 48.0 | 1.2 | 0.8 | 4.3 |
|  | ENSECAG00000036280 |  |  | 50.0 |  |  |  |
|  | ENSECAG00000018490 | ENSG00000127124 | HIVEP3 | 52.9 | 3.1 | 2.9 | 0.1 |
|  | ENSECAG00000016399 | ENSG00000021776 | AQR | 58.6 | 4.8 | 4.6 | 11.8 |
|  | ENSECAG00000008974 | ENSG00000114861 | FOXP1 | 59.3 | 1.1 | 1.4 | 7.5 |
|  | ENSECAG00000019813 | ENSG00000131196 | NFATC1 | 64.6 | 3.0 | 3.1 | 3.8 |
|  | ENSECAG00000020035 | ENSG00000101276 | SLC52A3 | 72.9 | 0.0 | 0.0 | 0.1 |
| Early response | ENSECAG00000000420 | ENSG00000172530 | BANP | -43.6 | 0.0 | 0.0 | 2.8 |
|  | ENSECAG00000008186 | ENSG00000067066 | SP100 | -34.5 | 22.2 | 25.6 | 12.5 |
|  | ENSECAG00000022473 | ENSG00000133816 | MAST1 | -25.5 | 4.8 | 5.1 | 13.0 |
|  | ENSECAG00000008259 | ENSG00000105613 | MASP1 | -23.8 | 0.0 | 0.0 | 0.6 |
|  | ENSECAG00000021049 |  | GDAP1L1 | -21.8 | 0.0 | 0.0 |  |
|  | ENSECAG00000010943 |  |  | -15.9 | 0.0 | 0.0 |  |
|  | ENSECAG00000012853 | ENSG00000189120 | SP6 | 34.5 | 0.0 | 0.0 | 0.1 |
|  | ENSECAG00000021446 |  | GRID1 | 36.0 | 0.0 | 0.0 |  |
|  | ENSECAG00000025011 |  | C11orf86 | 36.0 | 0.0 | 0.0 |  |
|  | ENSECAG00000016850 | ENSG00000001631 | KRIT1 | 36.7 | 4.7 | 3.8 | 11.8 |
|  | ENSECAG00000012027 |  | CACNA1E | 42.7 | 0.0 | 0.0 |  |
|  | ENSECAG00000041009 |  |  | 44.0 |  |  |  |
|  | ENSECAG00000023190 |  | MICAL2 | 44.3 | 0.0 | 0.0 |  |
| Mid response | ENSECAG00000018622 | ENSG00000068724 | TTC7A | -61.0 | 3.7 | 4.5 | 9.3 |
|  | ENSECAG00000015133 | ENSG00000242802 | AP5Z1 | -54.4 | 2.8 | 2.6 | 10.5 |
|  | ENSECAG00000019718 | ENSG00000157927 | RADIL | -54.4 | 0.0 | 0.0 | 0.3 |
|  | ENSECAG00000001342 | ENSG00000171222 | SCAND1 | -48.2 | 352.2 | 308.4 | 97.8 |
|  | ENSECAG00000016169 |  | COL6A6 | -46.0 | 0.0 | 0.0 |  |
|  | ENSECAG00000023646 | ENSG00000067208 | EVI5 | -45.7 | 0.2 | 0.2 | 3.8 |
|  | ENSECAG00000006979 | ENSG00000164574 | GALNT10 | -44.3 | 1.0 | 1.1 | 42.3 |
|  | ENSECAG00000024739 |  | CSTPP1 | -43.6 | 0.0 | 0.0 |  |
|  | ENSECAG00000024744 | ENSG00000149182 | ARFGAP2 | -43.6 | 68.3 | 51.9 | 41.5 |
|  | ENSECAG00000031172 | ENSG00000125968 | ID1 | -42.9 |  |  | 14.0 |
|  | ENSECAG00000008976 | ENSG00000159658 | EFCAB14 | -42.1 | 6.8 | 5.6 | 25.5 |
|  | ENSECAG00000000482 | ENSG00000099385 | BCL7C | -41.8 | 63.2 | 57.2 | 28.5 |
|  | ENSECAG00000037025 |  |  | -40.0 |  |  |  |
|  | ENSECAG00000012796 | ENSG00000150967 | ABCB9 | -39.0 | 0.3 | 0.2 | 1.0 |
|  | ENSECAG00000022834 | ENSG00000103091 | WDR59 | -37.4 | 3.4 | 3.0 | 14.8 |
|  | ENSECAG00000000291 | ENSG00000197943 | PLCG2 | -37.1 | 8.7 | 8.6 | 1.8 |
|  | ENSECAG00000014815 | ENSG00000266964 | FXYD1 | -36.7 | 1.0 | 0.5 | 0.1 |
|  | ENSECAG00000028572 | ENSG00000221946 | FXYD7 | -36.7 |  |  | 0.2 |
|  | ENSECAG00000035102 |  |  | -36.7 |  |  |  |
|  | ENSECAG00000043434 |  |  | -36.7 |  |  |  |
|  | ENSECAG00000015938 | ENSG00000149136 | SSRP1 | -36.2 | 4.3 | 3.5 | 153.8 |
|  | ENSECAG00000014550 | ENSG00000183020 | AP2A2 | 30.0 | 6.5 | 5.7 | 13.0 |
|  | ENSECAG00000010104 | ENSG00000120049 | KCNIP2 | 30.5 | 1.4 | 1.0 | 0.5 |
|  | ENSECAG00000020347 | ENSG00000120029 | ARMH3 | 30.5 | 0.6 | 0.5 | 13.0 |
|  | ENSECAG00000022559 | ENSG00000172500 | FIBP | 30.6 | 69.2 | 51.6 | 39.3 |
|  | ENSECAG00000031061 | ENSG00000175602 | CCDC85B | 30.6 |  |  | 100.0 |
|  | ENSECAG00000009095 | ENSG00000146083 | RNF44 | 30.8 | 122.5 | 121.1 | 11.0 |
|  | ENSECAG00000036484 |  |  | 30.8 |  |  |  |
|  | ENSECAG00000041727 |  |  | 31.4 |  |  |  |
|  | ENSECAG00000037030 |  |  | 33.1 |  |  |  |
|  | ENSECAG00000021031 | ENSG00000124181 | PLCG1 | 35.3 | 99.9 | 121.9 | 5.5 |
|  | ENSECAG00000042538 | ENSG00000113209 | PCDHB5 | 35.3 |  |  | 0.1 |
|  | ENSECAG00000019772 | ENSG00000196396 | PTPN1 | 36.0 | 14.2 | 19.0 | 21.8 |
|  | ENSECAG00000016862 | ENSG00000171612 | SLC25A33 | 36.4 | 2.9 | 4.7 | 9.0 |
|  | ENSECAG00000038761 | ENSG00000129968 | ABHD17A | 36.7 |  |  | 24.5 |
|  | ENSECAG00000015058 | ENSG00000163947 | ARHGEF3 | 40.0 | 7.7 | 13.5 | 3.8 |
|  | ENSECAG00000012813 | ENSG00000154743 | TSEN2 | 48.0 | 1.2 | 0.8 | 4.3 |
|  | ENSECAG00000036280 |  |  | 50.0 |  |  |  |
|  | ENSECAG00000018490 | ENSG00000127124 | HIVEP3 | 52.9 | 3.1 | 2.9 | 0.1 |
|  | ENSECAG00000016399 | ENSG00000021776 | AQR | 58.6 | 4.8 | 4.6 | 11.8 |
|  | ENSECAG00000008974 | ENSG00000114861 | FOXP1 | 59.3 | 1.1 | 1.4 | 7.5 |
|  | ENSECAG00000019813 | ENSG00000131196 | NFATC1 | 64.6 | 3.0 | 3.1 | 3.8 |
|  | ENSECAG00000020035 | ENSG00000101276 | SLC52A3 | 72.9 | 0.0 | 0.0 | 0.1 |

**Supplementary Table S7.** Animals recruited for the study.

| **ID** | **Sex** | **Age (y)** |
| --- | --- | --- |
| 1 | M | 3 |
| 2 | M | 3 |
| 3 | M | 2 |
| 4 | M | 2 |
| 5 | F | 3 |
| 6 | F | 2 |
| 7 | M | 2 |
| 8 | F | 2 |
| 9 | M | 2 |
| 10 | M | 2 |
| 11 | M | 2 |
| 12 | F | 2 |
| 13 | F | 2 |
| 14 | F | 2 |
| 15 | F | 2 |
| 16 | F | 2 |
| 17 | F | 2 |
| 18 | F | 3 |
| 19 | F | 3 |
| 20 | F | 2 |

**Supplementary Table S8.** Separate libraries for Illumina-index (the Px nomenclature in the Acyl column refers to the barcode used).

**Supplementary Table S9**. *In silico* results for the 5 enzymes considered, coupled with *Mse*I as companion enzyme. “Suitable fragments” are those composed by the enzyme couple within the decided length range. “Duplicate RAD sites” counts the number of sites that generate two suitable fragments.

|  | **AciI** | **AclI** | **AgeI** | **BsrFI** | **BstBI** | **MseI** |
| --- | --- | --- | --- | --- | --- | --- |
| Sites in equcab3 | 4327444 | 210713 | 70703 | 532609 | 151082 | 16877835 |
| Fragments (all) | 6176149 | 418370 | 138028 | 916561 | 300439 |  |
| Fragments (with MseI) | 1851605 | 207768 | 68086 | 385968 | 149530 |  |
| Suitable Fragments | 938748 | 91336 | 42344 | 253414 | 64274 |  |
| Duplicate RAD Sites | 55194 | 5971 | 3187 | 20142 | 4389 |  |

|  | Lab_ID | Sample_ID | AciI | Indexes |
| --- | --- | --- | --- | --- |
| Seq Library 1 | 1  2  3  4  5 | Bulk_T0_1  Bulk_T0_2  Bulk_T0_3  Bulk_T0_4  Bulk_T0_5 | T0_1_P7  T0_2_P8  T0_3_P9  T0_4_P25  T0_5_P26 | Index_2 |
| Seq Library 2 | 6  7  8  9  10 | Bulk_T30_1  Bulk_T30_2  Bulk_T30_3  Bulk_T30_4  Bulk_T30_5 | T30_1_P7  T30_2_P8  T30_3_P9  T30_4_P25  T30_5_P26 | Index_4 |
| Seq Library 3 | 11  12  13  14  15 | Bulk_T90_1  Bulk_T90_2  Bulk_T90_3  Bulk_T90_4  Bulk_T90_5 | T90_1_P7  T90_2_P8  T90_3_P9  T90_4_P25  T90_5_P26 | Index_5 |

**Supplementary Table S10.** Primer pair sequences used for RT-qPCR analysis with related amplicon length of the generating amplicons and accession numbers.

| Gene name | Primer forward | Primer reverse | Amplicon Length (bp) | Accession |
| --- | --- | --- | --- | --- |
| AP5Z1 | GGAGAGTCTGCTCCGACAGG | GGTGGCCGAGACGATAAGG | 148 | XM_023655186.1 |
| ARFGAP2 | TAGCCTACCAGGAGCTGCAA | TGCATTTCAGACAGCACCGA | 148 | XM_005598081.3 |
| ATP2A3 | GCAGCTGACCACCTCCTG | TGAGGTACACACGGGAGACT | 94 | XM_005597673.3 |
| EVI5 | AGCTGAGTCTCAATGTGCACT | CTGCTTCTCTCAGTTTCACAGC | 137 | XM_023641636.1 |
| GRID1 | ACCAAGCCATGGAATGGAGG | GTCTCACTGTAGGTCGTGCC | 146 | XM_023648767.1 |
| LHX1 | AAATGCAACCTGACCGAGAAG | CAGGTCGCTAGGGGAGATG | 123 | XM_023652963.1 |
| RADIL | AGCGCTGATGCCTTCAAATC | CTGGTAGATGCTGTCGTCCG | 126 | XM_023655188.1 |
| RGS19 | ACACAGGGCCAGAGGAGG | CGCCGCCGCTCTTCATTC | 139 | XM_023626983.1 |
| SCAND1 | GAGAAAGAGGAAGGAGCCGG | TCAGGGGTCGACGAGGAG | 71 | XM_001501876.5 |
| TTC7A | CGAGAGGGAGGAGGAGGTG | TTCAGATGCCGTGATGTCGT | 111 | XM_023619129.1 |
| HPRT (reference) | AATTATGGACAGGACTGAACGG | ATAATCCAGCAGGTCAGCAAAG | 121 | AY372182 |
| SDHA (reference) | GAGGAATGGTCTGGAATACTG | GCCTCTGCTCCATAAATCG | 91 | DQ402987 |

**Supplementary Figure S1.** Dendrogram showing cluster analysis of the libraries.

**Supplementary Figure S2.** Venn diagram clarifying the rationale behind this diversification in early and mid-term differentially methylated genes. Yellow colored area comprises the genes that are differentially methylated at least at T30 (early response); blued colored area comprises genes that are not differentially methylated at T30 (mid-term response).
